# Integrated child nutrition, parenting, and health intervention in rural Liberia: A mixed-methods feasibility study

**DOI:** 10.1371/journal.pone.0311486

**Published:** 2024-12-13

**Authors:** Sejla Isanovic, Musa Sanoe, Shelbie Wooten, Edward A. Frongillo, Aisha K. Yousafzai, Christine E. Blake, Mufaro Kanyangarara, Melanie Swan, Nicole Rodger, Miriam Murray, Leila M. Larson

**Affiliations:** 1 Department of Health Promotion, Education, and Behavior, Arnold School of Public Health, University of South Carolina, Columbia, South Carolina, United States of America; 2 Plan International, Plan International Liberia, Congo Town, Monrovia, Liberia; 3 Food and Agriculture Organization of the United Nations (FAO), Monrovia, Liberia; 4 Department of Global Health and Population, Harvard T.H. Chan School of Public Health, Boston, Massachusetts, United States of America; 5 Department of Epidemiology and Biostatistics, Arnold School of Public Health, University of South Carolina, Columbia, South Carolina, United States of America; 6 Early Childhood Development Network, Plan International, Plan International Global Hub, Woking, Surrey, United Kingdom; University for Development Studies, GHANA

## Abstract

In Liberia, children are exposed to multiple forms of adversity which can negatively impact their health and development. Research is needed to examine the feasibility and benefits of integrated interventions that can be incorporated into existing health delivery programs to simultaneously address low responsive stimulation, undernutrition, and infection. This study assessed the feasibility of an integrated intervention promoting psychosocial stimulation and improved child feeding by the provision of eggs and fish. The integrated intervention was incorporated into an existing government health program. Thirty female caregiver-child dyads were randomly selected from two rural communities in Liberia. Participants received fortnightly group parenting sessions and weekly eggs and fish designated for child consumption, for four weeks. Trained community health workers delivered the intervention. Assessments were conducted before and after the intervention using quantitative surveys and qualitative interviews. At baseline, we examined the home environment, caregiver-child interactions, diet, and infection control practices. At endline, we assessed the feasibility of the intervention. Descriptive analyses were conducted with quantitative data. Qualitative data were analyzed using conventional content analysis. Baseline findings indicated uncommon responsive parenting, inadequate early learning opportunities, high food insecurity, and high child morbidity. Mixed methods indicators of feasibility, including acceptability, adoption, and fidelity were high. Qualitative data from this feasibility study informed several future modifications to the program, including engaging fathers, supplementing group sessions with home visits, and broadening facilitator eligibility. This integrated intervention is feasible and can be incorporated into existing health programs to support early child development.

## Background

About 249 million children under the age of 5 years in low- and middle-income countries (LMICs) are at risk of not achieving their developmental potential [1]. These conditions can leave children unprepared for school, at higher risk of poor academic performance, and less able to cope with stress, all of which have long-term consequences for work productivity and overall well-being [1]. Children’s exposure to adverse conditions in low-income countries, particularly low levels of responsive parenting and early learning opportunities (henceforth referred to as responsive stimulation), poor nutrition, and high infection, can negatively impact their health and development [1]. Improving caregiver knowledge and skills to meet a child’s emotional, physical, and cognitive needs is crucial to children’s development, enabling them to reach their full potential. In areas with high burdens of poor responsive stimulation, malnutrition, and infectious diseases, such as malaria and diarrhea, leaving one or more of these conditions unaddressed may be a significant impediment and a missed opportunity to achieve an intervention’s full impact [2].

Previous studies have investigated the benefits of parenting interventions on child development [3] either alone or in combination with nutrition supplementation and nutrition education [4]. There is ample evidence demonstrating the benefits of responsive stimulation interventions on children’s cognitive, language, and motor development milestones [5]. Responsive stimulation interventions incorporating nutrition education were not found to have added benefits for child development [4, 6], whereas interventions that leveraged food provisions have resulted in additional improvements in development beyond responsive stimulation alone [7, 8]. These studies, however, have not investigated the provision of nutrient-rich animal-source foods and were constrained by small sample sizes [7, 8] and populations with limited generalizability [8, 9].

Addressing individual determinants of development may be effective in certain settings [6], but given the potential breadth of adversities faced by children living in resource-limited settings, addressing individual determinants may not be sustainable or sufficient to enable a child to reach their developmental potential. Several studies have explored integrated interventions targeting multiple determinants of child development. For instance, the WASH-Benefits trials conducted in Kenya and Bangladesh investigated the effects of interventions on water quality, sanitation, handwashing, and nutrition on child development [10, 11]. Moreover, the RINEW project in Bangladesh and interventions in Uganda used multiple components to enhance early child development [12–14]. While these studies provide insights into the benefits of integrated interventions, the specific focus on the incorporation of responsive stimulation, nutrition (particularly through the provision of nutrient-rich animal-source foods), and infection control in our study extends the existing body of knowledge. Furthermore, demonstrating that an intervention can be incorporated into an ongoing, established program is important to its scalability and longevity.

Limited evidence exists for the feasibility of an integrated (i.e., multi-input) and incorporated set of interventions that address the key determinants of development, namely responsive stimulation, nutrition, and infection. In the context of this study, and in alignment with the existing literature, the term “integrated” is used to mean a complex intervention addressing multiple determinants by combining intervention components [15, 16]; the term “incorporated” is used to indicate the full adoption of interventions into existing systems in one place [17]. Research to examine the feasibility–defined in this study as acceptability, adoption, and fidelity–of such an integrated intervention incorporated into an existing health delivery system is needed to inform future effectiveness trials.

Our study examined the feasibility of an integrated intervention promoting psychosocial stimulation and improved feeding through the provision of eggs and fish (nutrient-dense animal-source foods) provided to caregivers and their children living in rural Liberia. The study objectives were to 1) determine how the home environment, responsive caregiver-child interactions, diet (including animal-source foods), and infection control practices are experienced by caregivers of children aged 6–36 months and 2) assess the feasibility of the integrated intervention among caregivers and intervention facilitators.

## Methods

### Study design

Starting in February 2022, a four-week intervention was administered to participating caregivers and their children residing in two communities within rural Bomi County, Liberia. The study used a single-arm, mixed-methods design involving data collection at baseline and endline to assess the feasibility of the intervention; given the objective to assess feasibility rather than impact, a comparison arm was not needed. The intervention consisted of two components: fortnightly group responsive parenting sessions for caregivers and weekly provision of eggs and fish for children, integrated into the existing national disease prevention and treatment activities. The study was approved by the Institutional Review Boards of the University of Liberia–Atlantic Center for Research & Evaluation (reference number 21-12-299) and the University of South Carolina (reference number Pro00117979). Written informed consent was obtained from all participating caregivers, community health workers, and general community health volunteers.

### Participants and recruitment

The study was located in the North Western region in Liberia. Over half of the county’s population falls within the country’s lowest and second-lowest wealth quintiles. The 2016 Malaria Indicators Survey showed a malaria prevalence of 45% among children aged 6–59 months in the study area [18]. According to the 2019–20 Liberia Demographic Health Survey, the prevalence of anemia in children 6–59 months was high at 81%, 32% of children were stunted, 6% of children were malnourished, and 88% of caregivers reported using some form of violent discipline on their children [19].

The study’s sample comprised 30 caregiver-child dyads selected from two communities. The population for this study was female caregivers and their children aged 6–36 months. Female caregivers were eligible to participate if they had at least one child between 6 and 36 months and planned to reside in the community for the one-month duration of the study. The initial eligibility for female caregivers was ≥15 years old; however, our final sample did not include any caregivers under the age of 18. Therefore, all participants could provide informed consent for themselves. Women with cognitive and severe physical disabilities who were not able to implement the intervention practices were excluded from the study, as were children with developmental disabilities or known allergies to eggs or fish.

Community health workers from each community identified eligible caregivers and their children to participate in the intervention using a household listing of caregivers living in the community. The data collection team visited each community to offer a brief overview of the intervention to interested caregivers. Prior to enrollment, data collectors confirmed caregiver and child eligibility, read the consent form aloud to the caregiver, and obtained written informed consent from caregivers. Fifteen caregiver-child dyads were recruited from each community. Eligible caregivers residing in one community were selected using random sampling from the full list of eligible participants. In the other community, only 10 caregivers met the eligibility criteria; therefore, five additional caregiver-child dyads were randomly recruited from a neighboring area. This area was selected due to its similarities in socioeconomic and living conditions, ensuring consistency in the study’s participant profile.

In designing the study, the primary objective was to assess the feasibility rather than the effectiveness of the intervention. Hence, the estimation of a sample size of 30 dyads was grounded in qualitative methodologies, aimed at reaching thematic saturation concerning child diet, hygiene, and parenting practices [20]. This sample was deemed adequate for providing rich, contextual insights into the feasibility of the intervention’s components and the delivery process. At the outset, baseline data collection was performed on 30 dyads. The willingness to participate was universal, as no refusals were encountered. Follow-up data at the study’s conclusion were obtained from 29 caregivers; one was unavailable due to illness. One caregiver’s endline interview was not analyzable due to poor audio quality.

### Intervention

The integrated intervention was incorporated into the existing government disease prevention and treatment activities. Under the Liberia National Community Health Services Policy, insecticide-treated nets are provided to households nationally every three years. Community health workers act as delivery agents, performing monthly household visits to, among other activities, 1) conduct Integrated Community Case Management of child malaria, diarrhea, and pneumonia, and 2) promote the utilization of insecticide-treated nets, destruction of mosquito breeding sites, hygiene and cleaning of the home environment, and recognition of warning signs of malaria and other infectious diseases [21].

Given their responsibilities under the existing government-led health services policy, community health workers were recruited and trained to deliver the two additional intervention components. In both communities, the general community health volunteer (i.e., an informal health worker who provides services voluntarily and focuses on integrated community case management) [22] was trained alongside the community health worker on facilitating intervention delivery. Three members of the study team trained the community health workers and general community health volunteers (henceforth collectively referred to as community health workers) on intervention implementation. Training sessions involved didactic sessions, practice, and mock sessions. Study team members from Plan International Liberia monitored the intervention delivery, including the parenting sessions.

Community health workers distributed 14 eggs and 20 pieces of dried Bonny fish [6–11 grams each] to each caregiver every week for four weeks. Eggs and dry Bonny fish (*Sardinella maderensis*) were selected because they are typically consumed in Liberia and contain high concentrations of nutrients such as omega-3 fatty acids, vitamins B12 and D, iron, and zinc; no foodborne pathogens were previously detected in smoked Bonny fish samples collected from Ghana [23]. Caregivers were instructed to feed one egg every day of the week and a serving (i.e., around three pieces) of dried fish three days per week to the child participating in the intervention. Additional quantities of egg and fish were provided to be shared with other household members to discourage sharing of the food allocated to the child participating in the intervention. Before beginning the weekly food provisions, caregivers were provided with a cooking demonstration as a suggestion for how to prepare the egg and fish provided. The community health worker from each community conducted a locally relevant cooking demonstration on how to prepare the food. The demonstration consisted of mixing one serving of dried Bonny fish, pounded into dust, and one egg into fufu, a staple food made from cassava. The community health worker distinguished egg and fish preparation for children younger than 12 months and between 12 and 36 months. For children under 12 months, the fufu was cooked for a shorter period before mixing the egg and fish dust to maintain a liquid consistency. For children between 12 and 36 months, the fufu was cooked for an extended period to create a doughy consistency before adding the dried Bonny fish dust.

Each community health worker facilitated fortnightly group parenting sessions promoting responsive parenting through activities centered around the child’s developmental milestones. A total of two parenting sessions were provided to each group. The first session introduced caregivers to responsive parenting practices and informed caregivers of nutritious foods important to child growth and development. The second session engaged caregivers in problem-solving discussions and practicing skills to support healthy, holistic child development. All caregivers were instructed to attend and participate in both sessions. Caregivers were encouraged to bring their children to the sessions to practice the learned activities. The responsive parenting modules centered around engaging in two-way talk with the child, participating in age-appropriate and challenging play with their child, modeling love and respect, and following nutrition and hygiene practices (S1 Table). To ensure effective management of the sessions, caregivers in each community were split into two groups (i.e., group A and group B). Group A was composed of seven caregiver-child dyads, while group B was composed of eight caregiver-child dyads. Each group attended their respective sessions on consecutive days, scheduled in the mornings to accommodate caregivers’ availability. Further details of the intervention are provided in a template for intervention description and replication [24] guide (S2 Table).

### Data collection

The Medical Research Council framework was used to identify core elements of the complex intervention that were important for promoting psychosocial stimulation and improved feeding among caregivers to generate improvements in children’s development [25]. Data collected from caregivers at baseline assessed the context and culture of the two communities to inform the development of the implementation strategy [26]. Data collected from caregivers and community health workers at endline assessed the feasibility of the intervention.

A team of five local female data collectors with previous qualitative and quantitative data collection experience was assigned to collect either quantitative or qualitative data. Data collectors were not involved in the intervention implementation to maintain an objective perspective in data collection. In-country experts (MS and MM) reviewed the interview guides and questionnaires to ensure data collection instruments were appropriately tailored to the study context. Prior to data collection, data collectors underwent a rigorous five-day training led by the study’s principal investigator (LML). SI and MS oversaw data collection and held daily debriefings with the field research team to review transcript completeness and accuracy, as well as assess opportunities for improvement.

#### Quantitative data collection

Quantitative data were collected through questionnaires administered on a tablet at baseline and endline using KoboCollect v2022.1.2 [27]. Data were uploaded to a server daily. We used a structured multiple-choice questionnaire to obtain sociodemographic information on household composition, living situation, siblings, education, occupation, and income. Sociodemographic data were collected using questions adapted from the Demographic and Health Survey questionnaire [28]. A child dietary diversity score, minimum meal frequency value, and minimum acceptable diet value were created according to World Health Organization guidelines [29]. Food insecurity was measured using the Household Food Insecurity Access Scale [30]. We collected information on water, sanitation, and hygiene practices [31]. Morbidity in the last seven days was recorded through caregiver reports. Data collectors recorded the number of episodes and duration of diarrhea, bloody diarrhea, respiratory infection, fever, and vomiting. They also recorded any unplanned hospital or healthcare facility visits. Early learning opportunities were measured using the Tool for Early Learning [32], which measures the types of materials available in the home for the child to play with and interactions between caregiver and child (based on the Home Observation Measurement of the Environment [33]). Responsive feeding was assessed using a validated responsive feeding assessment [34]. Caregiver psychological distress was assessed using the Self-Reporting Questionnaire [35, 36]. The Caregiver-Reported Early Childhood Development Index (CREDI), a parent-report questionnaire, was used to assess child motor, language, cognitive, and socio-emotional development, and child mental health. The CREDI has been tested in over 26 countries [37], many of which are low-income. Questions were asked about how COVID-19 impacted the household, the caregiver, and the child. Referral procedures were in place for caregivers with high depressive symptoms or suicidality. For the child diet questionnaire, examples were adapted to use local foods. At the end of the four-week intervention, a quantitative questionnaire was administered to caregivers to collect information on feasibility, such as caregivers’ acceptability and adoption of the nutrition and responsive stimulation intervention components. Furthermore, at each parenting session, attendance was tracked by community health workers using attendance sheets.

#### Qualitative data collection

Qualitative data were collected using standardized interview guides at baseline and endline (S1–S3 Methods). Data collection was informed by the nurturing care framework, which emphasizes the importance of a supportive environment for early childhood development, focusing on key components such as health, nutrition, responsive caregiving, safety and security, and early learning [26]. This framework shaped our understanding of caregivers’ needs and the local context for the integrated intervention.

Caregivers were interviewed by trained data collectors at baseline, one week before the parenting intervention was implemented. Data collected from caregivers at baseline assessed caregivers’ perceived needs for an integrated parenting intervention and the culture and context of the two communities to inform the development of the implementation strategy (S1 Method) [26]. Following the parenting intervention, data collectors conducted endline interviews with caregivers to learn about their experiences participating in the intervention (S2 Method). Community health workers were also interviewed at endline to understand their perspectives on facilitating the parenting sessions. Interviews were conducted in an isolated area in the community. The duration of baseline and endline interviews averaged about 40 minutes per participant. Surveys and interview guides were administered in Liberian English and translated into American English for analysis.

#### Constructs of feasibility

Qualitative and quantitative data were used to understand the feasibility of the intervention, using constructs drawn from Pearson et al. [26] and Saunders et al. [38]. Three constructs were adapted to the current study to examine feasibility from the perspective of the participants (i.e., enrolled caregivers) and the facilitators (i.e., community health workers): acceptability, adoption, and fidelity (Table 1). Acceptability represented the extent to which participants were satisfied with the intervention and its alignment with their values and priorities. Adoption represented the extent to which participants actively engaged with, were receptive to, and applied resources and skills from the intervention. Fidelity represented the extent to which each intervention component was implemented according to the intervention protocol. The complete delivery of the intervention in this study entailed the addition of two components, the provision of fish and eggs and parenting sessions, built onto the standard package of infection control activities already established under the Liberia National Community Health Services Policy [21]. For the intervention to be considered ideal, the food provisions needed to be delivered to all participating caregivers each week on the scheduled day of delivery and according to the set quantities. In addition, the parenting sessions needed to be conducted fortnightly with the assigned group of caregivers, delivered in sequential order, and facilitated by the community health worker.

**Table 1:** Constructs of feasibility and type of information that contributed to each.

Construct	Respondent	
	Caregivers participating in intervention	Facilitators participating in intervention
**Acceptability**	• Experience participating in parenting sessions	• Reactions to parenting sessions from the local community
The extent to which participants were satisfied with the intervention and its alignment with their values and priorities.		
	• Provisions of food	• Facilitating parenting sessions
	• Content promoted in parenting sessions	• Content promoted in parenting sessions
**Adoption**	• Provision of egg and fish	• Practices promoted in parenting sessions
The extent to which participants actively engaged with, are receptive to and apply resources and skills from the intervention.		
	• Practices promoted in parenting sessions (changes in play and talk)	• Messages promoted in parenting sessions
	• Messages promoted in parenting sessions	
	• Attendance of sessions by caregivers	
	• Ability to reproduce practices promoted in parenting sessions at home	
**Fidelity**	• Timeliness and accuracy of required quantities of fish and egg deliveries to participating households	• Timeliness and accuracy of required quantities of fish and egg deliveries to participating households
The extent to which the intervention was implemented as planned.		
		• Delivery of content promoted in parenting sessions according to protocol
		• Timeliness in delivery of parenting sessions according to protocol

### Data analysis

#### Quantitative data analysis

Data were analyzed using SAS version 9.4 (SAS Institute, Cary, NC). Demographic and other variables of household, caregiver, and child characteristics were summarized descriptively and presented by timepoint (baseline and endline). Descriptive data were presented as count and percentage for categorical variables, mean ± standard deviation (SD) for normally distributed continuous variables, and median and quartiles (25^th^ and 75^th^) for non-normally distributed variables.

#### Qualitative data analysis

All interviews were audio-recorded on two devices and transcribed verbatim by an external transcription service [39]. Two members of the study research team (SI and SW) reviewed each transcript for accuracy. Data collectors completed field notes in English to provide details about the caregiver’s behavior during the interview and whether the caregiver understood the interview questions. Following each interview, data collectors recorded methodological observations and the quality of caregivers’ responses in their field notes. Field notes and observations were not used for interviews with community health workers; their interviews were focused primarily on their perspectives on the intervention’s effects and did not necessitate additional observational data.

The qualitative data analysis was conducted by two members of the study research team (SI and SW). Both researchers have experience conducting qualitative research. SI led the qualitative analysis under the guidance of the study’s principal investigator (LML). An inductive thematic analysis was conducted to assess the current state of parenting practices and health behaviors among caregivers of children between 6 and 36 months of age and the feasibility of the integrated intervention. This analytical method was selected to allow themes to emerge organically from the data [40, 41]. Transcripts were reviewed for accuracy, and field notes provided context on caregiver behavior and comprehension during interviews. Preliminary coding of the transcripts was conducted to build an initial codebook. Initial coding focused on explicit statements made by the participants. Examples of semantic-level coding include “handwashing before meals,” “child vaccination,” and “feeding practices.” To ensure coding reliability and the development of a robust codebook, 20% of the transcripts were randomly selected and independently coded by two researchers (SI and SW). Following independent coding, the researchers convened to compare findings, discuss discrepancies, and refine the codebook. This coding procedure continued until saturation was reached. Data saturation was determined when transcripts yielded no new codes or themes. After refining the codebook, SI and SW conducted another review of all transcripts to ensure that the updates in the codebook were consistently applied across all analyzed transcripts, maintaining uniformity in the analysis. Coded data extracts were sorted and collated into broader themes, using latent coding to create a list of themes and subthemes that captured the underlying meaning of the data. Examples of latent codes include “concerns about disease prevention” and “cultural practices influencing health behaviors.” Themes were reviewed and refined through team meetings. The final list of themes was validated by all coauthors to ensure interpretations were grounded in the data. NVivo (Version 12), a computer-based software for qualitative research, was used for systematic organization and retrieval of data, ensuring a transparent and organized analysis process. The final list of themes was vetted through peer debriefing and team consensus, ensuring clarity and coherence.

#### Inclusivity in global research

Additional information regarding the ethical, cultural, and scientific considerations specific to inclusivity in global research is included in the (S1 Questionnaire).

## Results

**Fig 1:**
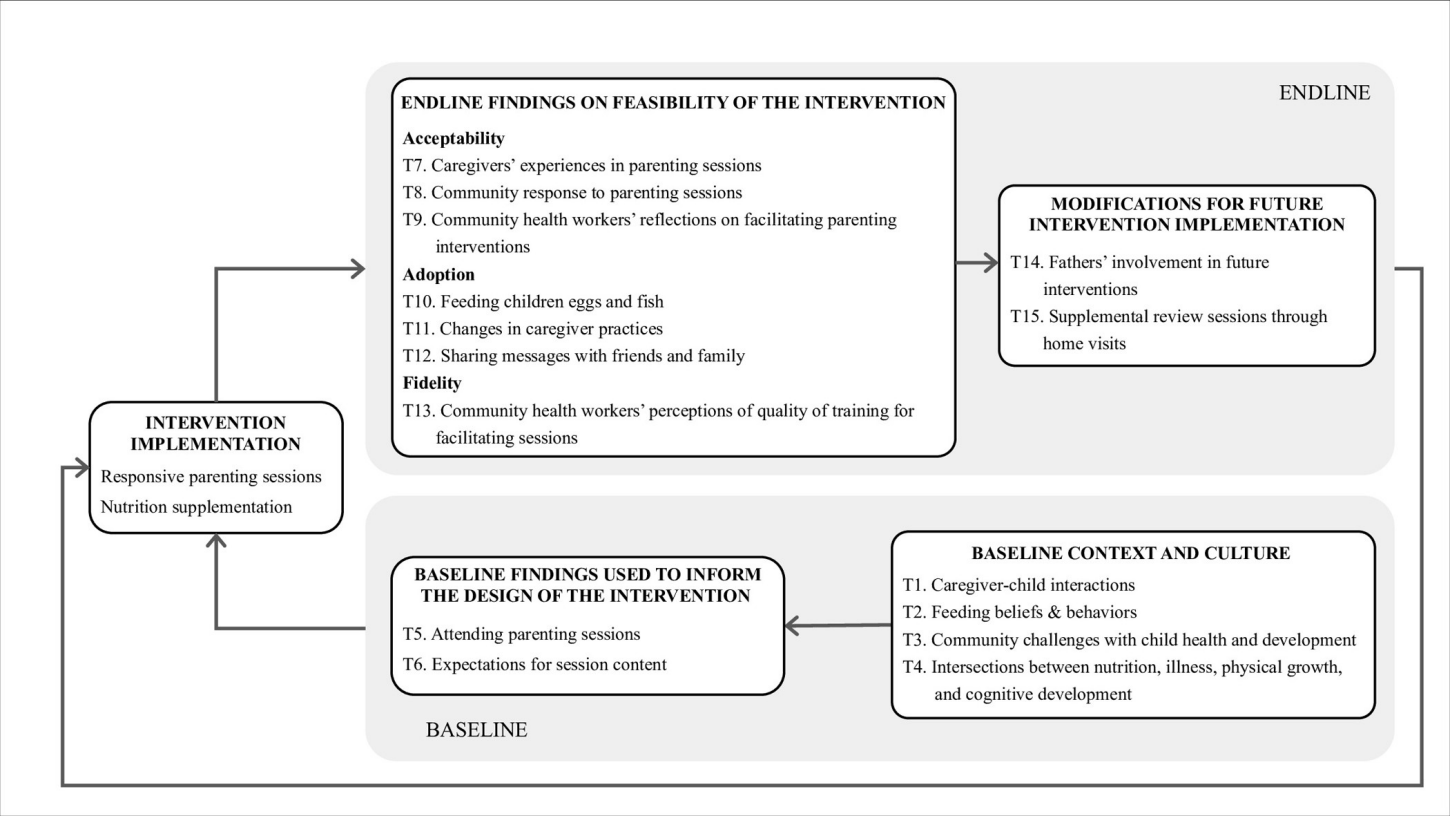
Organization of qualitative themes from baseline and endline qualitative interviews. Note. T, Theme.

Results were organized into four categories: 1) baseline context and culture, 2) baseline findings used to inform the design of the intervention, 3) endline findings on feasibility of the intervention, and 4) modifications for future intervention implementation (Fig 1).

### Baseline context and culture

The average household size in the two communities was about seven members (S3 Table). At baseline, over half of female caregivers had no formal education (Tables 2 and S3). Among those with children under 24 months of age, 60% were currently breastfeeding (Table 2). Disciplinary practices varied. Most caregivers reported explaining wrongdoings to their child or redirecting their attention; 43.3% of caregivers reported that they or another adult used verbal reprimands such as shouting, yelling, or screaming, and 33.3% reported using physical actions such as hitting or slapping. In terms of malaria prevention, many (76.7%) households had an insecticide-treated bed net, but only 21.7% of households utilized them the previous night. One-fifth of households relied on surface water as their main source of drinking water and a majority (86.7%) lacked basic sanitation facilities. Food insecurity was pervasive; 36.7% of households faced severe food insecurity with the remainder reporting moderate levels. Despite the widespread food insecurity, 80% of children met the minimum dietary diversity. The prevalence of childhood fever and cough in the past two weeks was high, with over 50% of children experiencing these symptoms. Responsive feeding was uncommon, evidenced by only 46.7% of caregivers reporting regularly engaging in responsive feeding behaviors and 80% reporting rarely allowing children to decide what to eat (S3 Table). Low early learning scores indicated limited early learning opportunities. For instance, less than 10% of caregivers reported having access to picture books and less than 5% reported reading and telling stories to their child. Detailed sociodemographic data, including information on water, sanitation, and hygiene (WASH), are provided in the (S3 Table).

**Table 2:** Demographic characteristics of the 30 study participants at baseline.

Characteristics	Number/number of responses (%) or mean +/- SD
**Sociodemographic**	
Mean caregiver age (years)	29.8 +/- 9.9
Mean child age (months)	18.1 +/- 9.1
Female child	14/30 (46.7)
Religion	
Christian	22/30 (73.3)
Islam	8/30 (26.7)
Female caregiver: no education	17/30 (56.7)
Female caregiver: regular job	27/30 (90)
Father: no education	16/30 (53.3)
Father: regular job	23/30 (76.7)
Monthly family income (Liberian dollars)	
<10,000	17/30 (56.7)
10,000-<50,000	13/30 (43.3)
**Insecticide-treated nets (ITN)**	
Household has ITN	23/30 (76.7)
Net obtained from government health facility	16/23 (69.6)
Net used last night	5/23 (21.7)
**Water, sanitation, and hygiene**	
Main source of drinking water for members of household	
Tubewell/borehole (improved drinking source)	24/30 (80)
Surface water (river, dam, lake, pond, stream, canal, irrigation channels) (no drinking water facilities)	6/30 (20)
Type of toilet facility	
Piped sewer system (improved sanitation facility)	1/30 (3.3)
Pit latrine (unimproved sanitation facility)	3/30 (10)
No facilities or bush or field (no sanitation facility)	26/30 (86.7)
**Food insecurity**	
Food insecurity score (range: 0–27)	11.2 +/- 3.8
(higher score indicates higher food insecurity (access))	
**Maternal psychological distress**	
Psychological distress score (range: 0–20)	2.9 +/- 2.3
(higher score indicates worse psychological distress)	
**Child discipline**	
Discipline score (range: 0–12)	3.6 +/- 1.3
(higher score indicates worse discipline actions by caregiver)	
**Child dietary diversity**	
Breastfed yesterday (children <24 months)	13/20 (65)
Meets minimum meal frequency (among children <24 months)	12/20 (60)
Meets minimum dietary diversity (≥5 of 8 food groups)	24/30 (80)
Child dietary diversity score (range 0–8)	5.5 +/- 2.0
**Child health**	
Diarrhea in the past 2 weeks	9/30 (30)
Fever in the past 2 weeks	17/30 (56.7)
Cough in the past 2 weeks	16/30 (53.3)
Difficulty breathing in the past 2 weeks	0/30 (0)
**Early learning opportunities**	
Early learning score (range 0–14); min = 1, max = 10	6.1 +/- 2.3
(higher score indicates better early learning environment)	
**Child development**	
Overall child development score (range: 0–108); min = 0, max = 55	26.1 +/- 16.9
Motor domain score (range: 0–40); min = 0, max = 30	15.3 +/- 9.7
Cognition domain score (range: 0–32); min = 0, max = 16	5.9 +/- 4.9
Language domain score (range: 0–39); min = 0, max = 11	3.3 +/- 3.8
Social emotional domain score (range: 0–23); min = 0, max = 11	5.2 +/- 2.7
**Child mental health**	
Child mental health score (range 0–9); min = 1, max = 6	2.5 +/- 1.3
(higher score indicates worse mental health)	

Additional data presented in S3 Table.

#### Theme 1: Caregiver-child interactions

Caregivers discussed their interaction with their child on a typical day. Only four caregivers discussed supervising their child when playing with others, and three reported engaging in play activities with their child. When caregivers were asked about their ability to engage in play or talk activities with their children given other competing priorities, such as completing household chores, most caregivers reported that they could not. Some (n = 5) caregivers explained that, while they worked, they would send their children to play with other children nearby, whereas other caregivers indicated they would put the child to sleep, breastfeed them, or occasionally offer them an object to play with (Table 3). Three caregivers reported interacting with their children through play or talk activities while working or doing chores.

**Table 3:** Qualitative reports from caregivers and community health workers at baseline.

Themes	Example quotations
**Baseline context and culture**	
*Theme 1*: *Caregiver-child interactions*	Interviewer: “Will you still have time to talk with [the child] even when you’re working… do you still have time to talk with him?” HH020 (caregiver, 31 years): “No. I can’t have time to talk with him, sometime he can come, but [I am] busy at work, […] I say he should go away to go play, sometime he will be going to cry, when he crying a lot, I say, ‘Go on outside.’ […] ‘Go play with your friends.’”
*Theme 2*: *Feeding beliefs and behaviors*	Interviewer: “Why? Why do you decide to give the child fish?” HH032 (caregiver, 35 years): “Because he get vitamin.” Interviewer: “So, why you think that food very important?” HH032 (caregiver, 35 years): “The thing that make the food very important, if can get it, it can make the child clever and make them to grow.” Interviewer: “So how you can prepared the rice for your child?” HH033 (caregiver, 38 years): “I can cook the rice with potatoes greens and Bonny for him.”
*Theme 3*: *Community challenges with child health and development*	HH038 (community health worker): “Parents have to put in different mechanism in their children’s growth. Coming to brain growth, they need a lot of different development food and those food are sometime around them, but they don’t see, they don’t see the need of using them. When we list down the category of food needed for the children brain growth or the children growth for education, [caregivers] will go and sell instead of eating it. Banana, they will find a banana in the community, get it ripe and then they will use it to sell. So, we notice that, those food are good for their growth but they are not materializing it.”
*Theme 4*: *Intersections between nutrition*, *illness*, *physical growth*, *and cognitive development*	Interviewer: “How does sickness affect her brain from developing fresh?” HH023 (caregiver, 19 years): “I don’t know.” Interviewer: “In your own thinking, how does the food the child eat, affect this sickness?” HH023 (caregiver, 19 years): “I don’t know.” Interviewer: “How food can make a child to get sick?” HH020 (caregiver, 31 years): “Yeah, some food not good for the child to give to them. When you give it to them, it make them sick. Not all the food good for the child.” Interviewer: “So when the child gets sick, it can affect their brain?” HH022 (caregiver, 43 years): “No.” Interviewer: “So the food that the child eating, it can get the child sick?” HH022 (caregiver, 43 years): “No. It can’t make the child sick.” Interviewer: “Do you think when the child gets sick it can affect their growing?” HH033 (caregiver, 38 years): “Yes.” Interviewer: “Do you think the sickness can also affect the child’s brain?” HH033 (caregiver, 38 years): “Yes, high fever can affect their brain.” Interviewer: “Do you think the food you giving your child is making them sick?” HH033 (caregiver, 38 years): “No.”
**Baseline findings used to inform the design of the intervention**	
*Theme 5*: *Attending parenting sessions*	Interviewer: “What you think would make it easier for you to go to the [parenting session]?” HH030 (caregiver, 26 years): “Um, well, if going, I can, I’ll give my child, I’ll give my child to my sister. I leave my child here, I come to the [parenting session].” Interviewer: “What do you think will be the easier way for you to go to the [parenting session]?” HH029 (caregiver, 24 years): “If like, if like distant, if I no have money I would not be able to go, but when I’m here, I’ll still go there, because I want it right near me, no transport.”
*Theme 6*: *Expectations for session content*	Interviewer: “I want you to tell me something about your feeling now in taking part in [parenting session] that will teach you about how to look after your children.” HH018 (caregiver, 34 years): “I will like for them to teach me how to take care of my children properly and also teach me about cleaning my yard, how to talk to my children so that they can’t feel bad because when your child talk to you and you shout at the child and say move from in front me, it making the child to feel bad.”

#### Theme 2: Feeding beliefs and behaviors

Caregivers’ decisions regarding feeding their children certain foods were categorized into two subthemes: perceptual features caregivers assigned to food and food access. Caregivers used foods’ perceptual features to identify the health and nutritional value of the food. Food access consisted of societal and environmental forces that enabled or hindered caregivers from feeding their children specific foods. Caregivers regarded foods such as eggs, fish, rice, and plantains as healthy. They viewed these foods as necessary for their child’s physical and cognitive development, referencing the food’s nutritional content (Table 3). For example, one caregiver described choosing fish for its high protein content, perceiving it as vital for child growth and brain development. Societal and environmental forces affecting food access included limited availability of certain foods in local markets and fluctuating prices that made nutritious foods like eggs unaffordable for many, while fish, being more readily available and affordable, was a common inclusion in children’s diets.

When caregivers were asked about the child’s food preparation (i.e., food items and preparation practices), about one-third (n = 11) reported feeding the child rice. The preparation of rice differed among caregivers. Some described ensuring the rice was cooked until the texture was soft to suit the children. Other caregivers reported feeding their children dry rice, a typical Liberian dish cooked with okra and fried fish. Fish was also frequently identified as a staple food provided to children; caregivers described adding raw or dried fish to rice and soup dishes (Table 3).

#### Theme 3: Community challenges with child health and development

Community health workers identified multiple challenges that impacted children’s health and development within their communities. They described caregivers as having little knowledge of nutrition and child feeding practices and were, therefore, not feeding their children adequate amounts of diverse foods (Table 3). Additionally, reports from community health workers indicated caregivers struggled to dedicate time to engage with their children in play activities due to prioritizing other responsibilities, such as household chores.

#### Theme 4: Intersections between nutrition, illness, physical growth, and cognitive development

Caregivers were asked to describe their child’s health, particularly whether they considered the effects of the child’s nutritional intake on illness susceptibility, physical growth, and cognitive development. Likewise, caregivers were asked about an illness’s impacts on their child’s physical growth or cognitive development. Two caregivers were unsure whether connections exist between nutrition, disease, physical growth, and cognitive development (Table 3). Several caregivers (n = 7) recognized the links between the foods children were fed and their physical growth, but most did not identify a connection between nutrition and illness or between nutrition and cognitive development. Almost half of caregivers (n = 13) agreed that illness might affect their child’s physical growth, but only six believed their child’s cognitive development could also be affected.

### Baseline findings used to inform the design of the intervention

The intervention design was informed by themes from baseline findings on caregivers’ barriers and facilitators to attending parenting sessions, as well as their expectations for session content. The themes informed two components of the intervention: fortnightly group responsive parenting sessions for caregivers and weekly provision of eggs and fish for children (Fig 1).

#### Theme 5: Attending parenting sessions

Caregivers’ responses indicated that the best time to participate in the parenting sessions would be in the morning to prevent scheduling conflicts with their daily activities. Potential reasons for not attending the parenting sessions primarily involved caregiving assistance needed for family members or children not involved in the sessions as well as any travel-related costs if the sessions were held in a nearby community (Table 3). Caregivers reported a preference for attending sessions near their community and indicated they were willing to participate in the sessions so long as they found them valuable for promoting their children’s growth and development.

#### Theme 6: Expectations for session content

While most caregivers did not specify which parenting skills they wished to improve, four caregivers indicated they hoped to improve specific techniques, such as safe food preparation and feeding practices, to minimize the frequency and severity of child morbidity (e.g., diarrhea, fever). Some (n = 5) also expressed interest in learning about environmental sanitation around their homes to keep their children healthy. Additionally, caregivers considered interacting with their children through talk an important skill to develop for healthy communication (Table 3).

These baseline findings guided the design of the content promoted in the parenting sessions, including the images depicted in the posters and handouts, the playthings used during session demonstrations and activities, the language and speech patterns used in the suggested script and dialogue for each session, the timing of the sessions, and the cooking demonstration (Fig 1).

### Endline findings on feasibility of the intervention

#### Acceptability

All caregivers found that the type of fish was acceptable to feed the participating child. Caregivers considered the number of eggs and fish delivered sufficient to feed the participating child each week (96.6%) (Table 4).

**Table 4:** Feasibility results from quantitative endline surveys.

Variables	Number/Number of Responses (%) or Mean +/- SD
**Acceptability**	
*Nutrition intervention*	
Considered quantity of egg and fish delivered sufficient	28/29 (96.6)
Type of fish was acceptable (yes)	29/29 (100)
**Adoption**	
*Nutrition intervention*	
Fed child at least one egg in the past week	29/29 (100)
How many times in the past week fed child eggs provided	
Less than 3 times/week	5/29 (17.2)
3 times/week	17/29 (58.6)
7 times/week	7/29 (24.2)
Egg never shared with others	29/29 (100)
Fed child at least one fish in the past week	29/29 (100)
How many times in the past week fed child fish provided	
Less than 3 times/week	8/29 (27.6)
3 times/week	14/29 (48.3)
7 times/week	7/29 (24.1)
Fish never shared with others	29/29 (100)
*Responsive stimulation intervention*	
Number of sessions attended	
One session	1/29 (3.5)
Two sessions	28/29 (96.5)
Interested in attending more sessions if had the opportunity	
Yes, very much	29/29 (100)
Number of messages recalled	
0 messages	1/29 (3.5)
4 messages	1/29 (3.5)
5 messages (all)	27/29 (93.1)
Able to do any of the following: feed child animal-source foods, 3 meals + 2 snacks, 4–8 handfuls of cooked food at each meal	28/29 (96.6)
Able to wash hands (and child’s) with soap and water before touching food and after latrine use	29/29 (100)
Able to provide child with stimulating play objects	29/29 (100)
Able to sing and talk with children and watch and listen to child’s sounds	29/29 (100)
Able to show love and respect to your partner and children	29/29 (100)

Additional data presented in S4 Table.

#### Theme 7: Caregivers’ experiences in parenting sessions

When asked about their thoughts on the information delivered at the parenting sessions, caregivers (n = 12) provided examples of content they enjoyed learning, such as safe food preparation, child nutrition, child feeding practices, sanitation and hygiene, and responsive communication through play and talk activities. Caregivers reported that they enjoyed the structure of the sessions and found the delivery of the material engaging. Community health workers shared reports from caregivers about their experiences during the sessions, stating caregivers’ appreciation of the informative discussions for providing a strong understanding of the content. Caregivers found the parenting sessions valuable in providing the knowledge and skills needed to care for their children effectively. During the month-long intervention, caregivers shared improvements in their child’s physical health and motor skills. They attributed these changes to the activities learned in the parenting sessions and the additional food provided (**Table 5**). Witnessing the intervention’s benefits enabled caregivers to see its value for their children’s health and development, as well as for future generations. They all reported interest in attending more sessions if they had the opportunity.

**Table 5:** Qualitative reports from caregivers and community health workers informing endline findings of feasibility and future modifications.

Themes	Example quotations
**Acceptability**	
*Theme 7*: *Caregivers’ experiences in parenting sessions*	Interviewer: “Tell me, what’s a message that you remember?” HH020 (caregiver, 31 years): “The way how they teach us how to take care of ourself, we the parent, to love ourself, our very self. […] to love our children, we should show love and respect to our loved one and our children. And how to talk and play with our children. […] And we should know the type of plaything to give to children when they are in need of playing. And before giving food to children or before feeding children, they should wash their hands.” Interviewer: “What is your opinion about the knowledge and skills shared with caregivers through their parenting program?” HH038 (community health worker): “I believe that [the caregivers] all understood well because the knowledge we share with them, we all interacted with them.” HH033 (caregiver, 38 years): “[The parenting sessions] teach me how to hold my child. Give me more sense, more idea. I like it. How they teach me, I like it a lot, and I want it to continue more, because it gave me more idea how to take care of my child.” Interviewer: “Please tell me how the workshop help you.” HH017 (caregiver, 23 years): “The [food provided] make my child body healthy. It make my child brain grow. Since I started giving the egg, the body was alright. It made the body healthy and good.”
	Interviewer: “In your opinion, what did the caregivers think of the parenting program?” HH036 (community health worker): “From the day we start teaching them, [caregivers] are saying this skill and knowledge, they value it.”
*Theme 8*: *Community response to parenting sessions*	HH038 (community health worker): “When we started the, the other sessions, we only noticed [caregivers] coming for those who are part of the program but in two to three weeks, so many community members were coming in. So, my question was, who invited you? Some of them said, we, we heard our aids, our friends to us, and we saw it interesting.”
*Theme 9*: *Community health workers’ reflections on facilitating parenting intervention*	HH036 (community health worker): “This program is very helpful to our children. As I’m speaking now, there are some children who was not even able to identify object or color before now. They can identify those. Through the children from, um, one year to three years now, they can identify color. But it’s just that some of them don’t talk. They don’t call the name. But when you, you show the picture to them, they just, they can point at the picture. They can point at the picture. Then the parents help to talk.” HH038 (community health worker): “When go into the session, we see parent playing with their children, we see parent providing materials. When they got to know that they don’t have to go, you don’t have to go to the market to buy play toys for your child. Toys are in the community. You look for some cups and put rock in it, they will play. So parents get to know that. They get to know that, those things are very useful.” HH038 (community health worker): “This program is very, very good because we did not grow up that way. We saw our parents, we went to school, our parents never have time for us. And we seen it on a daily basis in those villages and towns. […] It is very harmful for the children. […] And their brain will not grow the way it’s supposed to grow.”
**Adoption**	
*Theme 10*: *Feeding children eggs and fish*	Interviewer: “Why you feed the child with the egg and fish they give you?” HH014 (caregiver, 18 years): “Well, I want for my child to be strong and my child brain will grow and to be smart. So that they gave it to me and I gave it to them.” Interviewer: “Did you feed both, you getting both the egg and the fish?” HH014 (caregiver, 18 years): “Yeah, the egg and the fish, I get all two.”
*Theme 11*: *Changes in caregiver practices*	Interviewer: “Which [of the five messages] was hard for you to do?” HH010 (caregiver, 25 years): “Play, playing with child…” Interviewer: “So how it hard?” HH010 (caregiver, 25 years): “How it hard to me? Because some time, I can be busy. When I’m working like that, I can be busy. So, for me to say how it can be hard. But for the playing with her, I can … I understand the good so I can do what I can- I can’t miss it.”
	Interviewer: “So how do you think the things you learnt in this meeting can improve your child growing tall or activity of the child?” HH008 (caregiver, 18 years): “Yeah. Like the, the playing, because as you’re playing with the child, talking with the child, they will- it will make the child grow. [The playing and talking] will make [them] understand what you’re doing.”
*Theme 12*: *Sharing messages with friends and family*	Interviewer: “The things that they doing now that you told them about and things that they were doing before, what’s the difference now?” HH019 (caregiver, 25 years): “[The caregivers] can play with their child now. Difference is that [caregivers] can do good thing for that child, they can play with their children, they can take care of their children, they can wash their children hand before they eat.”
**Fidelity**	
*Theme 13*: *Community health workers’ perceptions of quality of training for facilitating sessions*	Interviewer: “What did you think about the quality of training you received on the parenting program?” HH036 (community health worker): “Uh, the training that I took, that training was very good, and it helped me to learn more about the children. And when I came back in my community, I taught my community people too. […] All of the training that undertake, uh, we undertake to come and train our, uh, caregivers, everything was useful.”
**Modifications for Future Intervention Implementation**	
*Theme 14*: *Fathers’ involvement in future interventions*	Interviewer: “Do you think [the father] should take part in the [parenting sessions] with you, or they should have different [sessions] for them?” HH005 (caregiver, 24 years): “They can have different [sessions].” Interviewer: “So, why you want [fathers] to have different [sessions]?” HH005 (caregiver, 24 years): “Because […] what the [session facilitators would] show me, I would already know it. I already know how to take care of child, already know how to maintain my child, to feed my child.” HH038 (community health worker): “We come home, we want our wives to make us water to take bath, want our wives to give us water to drink, forgetting to know that those [caregivers] need time. [Caregivers] need to play with their children. So if [fathers] are educated on those things too, we will find a good or good result in our daily activities with parents taking care of their children.”
*Theme 15*: *Supplemental review sessions through home visits*	Interviewer: “Do you think is important for the community health [worker] to always come to your home, to review the material you learn in the [parenting sessions]?” HH001 (caregiver, 31 years): “Yes.” Interviewer: “Why?” HH001 (caregiver, 31 years): “Why I say, because if the [community health worker] coming, or they come to [review with] us, we’ll still be learning more. We not forget.”

Caregivers found the visual aids (i.e., posters, handouts, and pictures) helpful in encouraging interactions with their child during play and talk activities, and in relaying key messages about appropriate feeding practices and caregiver-child interactions. Additionally, caregivers appreciated the comprehensive sessions and the use of demonstrations, explanations, and discussions to convey the content. Community health workers were pleased with the content and messages promoted in the parenting sessions. One community health worker stated that each session presented relevant information concerning caregivers and children, and that the delivery of the sessions allowed for productive discussions (Table 5).

#### Theme 8: Community response to parenting sessions

Community health workers reported that the intervention received positive reactions from the community. One community health worker described an influx of caregivers interested in participating in the second parenting session after hearing about the first session from participating caregivers (Table 5).

#### Theme 9: Community health workers’ reflections on facilitating parenting interventions

Community health workers expressed their eagerness to continue the parenting sessions after witnessing the impact that just two sessions had on caregiver knowledge and caregiver-child interactions. They praised the sessions for their practicality and sustainability, using an example of caregivers sourcing local, everyday materials from the community to create children’s playthings. One community health worker contrasted parenting practices typically seen in these communities with those illustrated in the sessions, emphasizing the need for parenting, nutrition, and health promotion practices (Table 5).

#### Adoption

Endline quantitative data indicated that all caregivers fed their children at least one egg and one piece of fish in the previous week (Tables 4 and S4). The majority fed their child eggs (82.8%) at least three times and fish at least three times (72.4%) in the previous week. About 24% of caregivers reported feeding their children eggs and fish every day of the week. Almost all caregivers (96.6%) indicated their ability to feed children eggs and fish. When feeding children, all caregivers reported that they could follow hygienic practices. Quantitative findings indicated that almost all caregivers attended all responsive stimulation intervention sessions (96.5%). Furthermore, 93.1% of caregivers could recall the five key messages taught during the parenting sessions and indicated that they practiced the activities from the key messages at home (Table 5). The five key messages of the parenting sessions were: 1) “Love”: Emphasizing the importance of showing affection to children to make them feel secure, 2) “Talk”: Focusing on the significance of speaking to infants and young children for brain development and language skills, 3) “Play”: Highlighting play as a critical element in all domains of a child’s development, including physical, cognitive, social-emotional, language, and executive function skills, 4) “Wash”: Underlining the importance of handwashing for parents and children to maintain cleanliness and hygiene, and 5) “Food”: Stressing the value of nutritious foods, especially animal-source foods, in the child’s brain development and overall health.

#### Theme 10: Feeding children eggs and fish

Caregivers frequently remarked on the effects they observed from feeding their children the eggs and fish provided by the intervention. All caregivers attempted to feed their children eggs and fish, and most indicated their children enjoyed the food. Caregivers reported that the food allocated to the child participating in the intervention was not shared among other family members (Table 5). The preparation of the food varied among households. Some caregivers followed the meal preparation demonstrated by the health worker at the cooking demonstration, while others prepared the eggs differently, either boiling or frying them instead of mixing them into fufu.

#### Theme 11: Changes in caregiver practices

The parenting sessions emphasized five messages (love, talk, play, wash, feed) for caregivers to follow to promote healthy growth and development in children. At endline, caregivers described the extent to which they implemented the five messages. Most caregivers indicated that the five messages were easy to implement. All caregivers reported being able to practice play and talk activities with their children. Several (n = 6) caregivers described some difficulties they faced, such as locating appropriate items in the community for their child to play with or finding time to practice play activities (Table 5). Endline interviews indicated that community health workers also adopted the messages and practices from the parenting sessions. They described applying the skills learned from facilitating the intervention at home, including following healthy feeding practices by providing their children with a diverse range of foods to support healthy growth and development.

Following the intervention, caregivers reported increasing interactions with their child through play and talk activities. Roughly one-third of the caregivers (n = 11) reported using play and talk as nonviolent discipline methods when their children were upset rather than responding with physical punishment (e.g., shouting, spanking). Caregivers found that the parenting sessions helped them understand the importance of utilizing these activities daily to encourage physical and cognitive growth and development, and to develop stronger connections with their children through communication (Table 5).

#### Theme 12: Sharing messages with friends and family

Most caregivers (n = 25) shared intervention-related content with their partners and community members. Community members were often nearby caregivers who did not participate in the intervention. Caregivers reported receiving positive responses from those with whom the messages were shared, indicating that the caregivers who could not participate in the intervention were receptive to the information and willing to apply the skills in their households (Table 5). Caregivers reported using two of the visuals received during the parenting sessions (i.e., “The Food Groups” handout and “The 5 Messages” handout) to convey the material to community members who were not participating in the intervention.

#### Fidelity

Required quantities of fish and eggs were delivered to all participating households at the start of each week. Parenting sessions were facilitated by community health workers and held fortnightly, in the correct order, in both communities.

#### Theme 13: Community health workers’ perspectives on quality of training for facilitating sessions

Community health workers reported that the training sessions they attended were helpful in implementing the intervention. They indicated that the training sessions provided them with the knowledge and skills to effectively communicate each session’s topic to caregivers (Table 5). Community health workers recognized the importance of the intervention in enhancing child development and caregiver education, indicating that the intervention was a worthwhile addition to their existing duties. The integration of the intervention was deemed manageable and was seen as complementing their ongoing community health initiatives.

### Modifications for future intervention implementation

The information collected from community health workers and caregivers about the intervention components informed potential modifications to future interventions, namely the engagement of fathers in parenting sessions and the inclusion of one-on-one review sessions between group sessions. Reflections from the study team informed a potential modification to the requirements for the role of intervention delivery agent.

#### Theme 14: Fathers’ involvement in future interventions

Caregivers were asked about the potential benefits and usefulness of fathers’ involvement in future interventions, and all agreed on the importance of including fathers in the sessions. Most caregivers preferred the parenting sessions to be joint with both the female caregiver and father, whereas a few (n = 3) thought it best to have separate sessions. Caregivers viewed the father’s engagement in the sessions as necessary to effectively implement parenting skills. Those in favor of joint sessions described the cooperative environment that having both parents at the sessions could build, referencing one of the five messages of learning to show love and respect to one’s partner. Caregivers who preferred separate sessions indicated that knowledge and skill sets related to parenting differed between roles; therefore, the sessions would better cater to each parent if held separately (Table 5). Caregivers who favored separate parenting sessions described some difficulties that attending joint sessions would present. One caregiver stated she would not be able to participate in the same session as the child’s father because she would be responsible for watching the children. Another caregiver indicated a preference for the intervention sessions to be held separately, while encouraging female caregivers and fathers to share what they learned with each other and reflect on their experiences from each session.

Community health workers shared similar views on fathers attending subsequent sessions. They believed that including fathers in joint sessions with caregivers would have a greater impact on the children if both parents supported one another in learning how to adopt the skills and implement the play and talk activities together. One community health worker suggested that involving and educating fathers could benefit caregivers if the fathers understand the importance of caregivers dedicating time to play and talk with their children (Table 5).

#### Theme 15: Supplemental review sessions through home visits

While recall of the messages promoted in the parenting sessions was high, caregivers supported the idea of implementing review sessions through home visits. Caregivers indicated that the review sessions would help them better retain the information learned during the group sessions (Table 5).

Lastly, the study team noticed that, while community health workers were willing to facilitate the parenting sessions, some struggled to effectively engage caregivers in a group setting. For future implementations of similarly integrated interventions, we recommend modifying the requirement for community health workers to run the parenting sessions and instead allow any interested, well-respected, and able community member (including the community health worker) to be trained and assume the role of intervention delivery agent.

## Discussion

An integrated nutrition and responsive stimulation intervention was delivered to 30 female caregivers and their children aged 6–36 months in rural Liberia. The intervention was innovative for two reasons. First, it combined intervention components to address multiple determinants of child development jointly. Second, the combined intervention components were incorporated into an established health delivery system. The incorporation of the intervention into the existing health system was achieved by leveraging the infrastructure of the Liberia National Community Health Services Policy, through which community health workers were already delivering the national policy’s infection control program. The community context indicated little responsive parenting and few early learning opportunities, high food insecurity, and high child morbidity, but a strong willingness to participate in a nutrition-and-parenting intervention. Overall, the intervention demonstrated the potential to benefit caregiver and child outcomes. The group parenting intervention, coupled with the delivery of eggs and dry fish for child consumption, demonstrated the potential to recruit and retain participants. Furthermore, the acceptability, adoption, and fidelity of the intervention components were high.

The parenting session delivery model was successful in this setting; caregivers and community health workers enjoyed the sessions and found them valuable. The structure of the parenting sessions used active coaching, a behavior change technique that promotes change in knowledge and practice [42]. After community health workers demonstrated an activity, time was allocated for caregivers to practice play activities with their children and learn by observing other caregivers, as has been done in previous successful programs [43, 44]. An active coaching format enables a supportive environment for caregivers to engage with content and one another through practice and discussion [42]. Multiple behavior change techniques were included to improve the delivery of the content, including visual aids, dialogue, demonstrations, problem-solving, practice, and social support. The supportive and engaging delivery model, in combination with context-relevant adaptations to the program, likely contributed to caregivers’ acceptance of the content and enabled community health workers to facilitate productive discussions.

Consideration of the limited resources accessible to caregivers and children in each community was paramount in implementing a feasible intervention; therefore, community health workers guided caregivers in sourcing local, everyday materials from the community to create children’s playthings. Jeong et al. found similar results in Mozambique, where caregivers and children enjoyed learning play and talk activities using locally constructed playthings [45]. There was no reported sharing of the food designated for the participating child, likely because the study provided additional quantities of eggs and fish to each participating household; similar findings have been reported in other food provision studies [46]. Regarding the sustainability and scalability of providing eggs and fish, the intervention’s feasibility was bolstered by establishing a local supply chain. This supply chain leveraged existing market structures and local producers. Its long-term sustainability, however, requires further evaluation, including assessment of market dynamics and community engagement, which needs to be evaluated in a larger effectiveness trial.

The community can play an important role in the success of an intervention. The study team involved key stakeholders from each community before beginning the intervention. Caregivers witnessed improvements in their child’s development and attributed this progress to their use of the knowledge gained and practices learned during the intervention. These observations prompted them to share the messages from the sessions with their peers, which increased community awareness of the importance of the intervention. Other behavior change studies have reported similar findings, showing an increase in local awareness of early childhood development through the sharing of messages by caregivers who participated in interventions [47–49].

Participating female caregivers indicated that fathers would benefit from attending similar parenting sessions. Therefore, future interventions may include components of father involvement in separate and joint sessions with female caregivers, depending on the content and context, given the influential role both female and male caregivers have in decision-making regarding infant and young child feeding [50]. In Vietnam, an intervention examining the effects of fathers’ involvement on infant development found that increased quantity and quality of fathers’ participation significantly impacted early childhood development [51]. Studies in Rwanda strengthen the support for father engagement, reinforcing its importance in encouraging male caregiving and promoting equality in household roles [52, 53]. A parenting intervention conducted in Kenya demonstrated that positive involvement by fathers, through interpersonal support to caregivers, was associated with improvements in childhood development; however, the study reported low levels of father participation [54]. Therefore, consideration of strategies best suited for motivating and encouraging fathers to participate in parenting sessions from the outset is critical.

This context underscores the need for careful selection and training of facilitators. Community health workers were not always the most appropriate delivery agents for the intervention, which involved delivering information and facilitating activities within a group setting–a responsibility that required a specific skill set and time commitment. The effectiveness of a facilitator in this intervention hinges on key skills such as strong communication, adept group facilitation, and empathy. Community health volunteers, often in informal roles, can be highly effective due to these skills and their strong community connections. Our findings suggest the importance of selecting facilitators based on such competencies for successful intervention delivery. Community health workers may also face more systemic and long-term challenges, such as high work burden, inadequate supervision, or onerous payment structures and delays [55]. The involvement of community health workers, however, is integral to the intervention’s incorporation into the existing health system. Therefore, establishing a liaison between the community health worker and the intervention facilitator, should someone besides the community health worker assume this role, is essential. Plan International Liberia, a non-governmental organization, acted as a key partner for this intervention, working in close collaboration with community health workers to support the implementation and facilitation of the intervention. Their involvement was instrumental in supporting this type of structure and ensuring effective delivery of the program.

Being a feasibility study, the sample size was small and limited to two communities in one county, no comparison arm was used, and the duration was limited to one month. Although the duration was short, the parenting sessions represented the range of skills that will be required of facilitators for the full parenting curriculum (i.e., didactic sessions, engaging caregivers in discussion, problem solving with participants, demonstrating to caregivers, and coaching as they practice activities with their child). Furthermore, the study established a supply chain for fresh eggs and dry fish. Participants might have provided socially desirable responses, given the sensitive nature of parenting practices and health behaviors. To mitigate this, data collectors were trained to create a non-judgmental environment and reassure participants that their responses were confidential and that there were no right or wrong answers. While the generalizability of the findings may be limited due to the small geographic focus of this study, the feedback we received from the caregivers and community health workers proved valuable, as their views, whether shared by others in the community or not, offer critical insights into what needs to be addressed in the content design and delivery for the effectiveness trial. This comprehensive approach ensures that the intervention remains responsive to the nuanced needs of the community. Changes in individual caregiver or child outcomes were not measured, as this was not an objective of the study, but the data on feasibility will be used to design a suitably large effectiveness trial in which the impact on individual outcomes will be tested.

## Conclusion

This integrated and incorporated nutrition and responsive stimulation intervention delivered to female caregivers and their children aged 6–36 months was feasible, demonstrating appropriate acceptability, adoption, and fidelity. Caregivers, community health workers, and general community health volunteers found the content and delivery of the intervention acceptable, and caregivers reported understanding the importance of utilizing activities from the parenting sessions to encourage their child’s development. Implementation of the study helped identify opportunities for improvement in intervention design and delivery. These findings will inform the design of an effectiveness trial and guide future studies aiming to deliver meaningful and sustainable benefits for children’s health and development. Comprehensively addressing the adversities faced by children in resource-limited settings, such as suboptimal responsive stimulation, poor nutrition, and high infection, could lead to sustained benefits for children and their caregivers.

## Supporting information

S1 Method Baseline qualitative interview guide for caregivers (Liberia English). (DOCX)

S2 Method Endline qualitative interview guide for caregivers (Liberian English). (DOCX)

S3 Method Endline qualitative interview guide for community health workers and general community health volunteers (Liberian English). (DOCX)

S1 Table Responsive parenting sessions. (DOCX)

S2 Table Template for intervention description and replication checklist. (DOCX)

S3 Table Demographic characteristics of study participants at baseline. (DOCX)

S4 Table Results from quantitative endline survey. (DOCX)

S1 Questionnaire Inclusivity in global research questionnaire. (DOCX)

S1 File Deidentified baseline database. (XLSX)

S2 File Deidentified endline database. (XLSX)
