# Supplementary material for: Integrated child nutrition, parenting, and health intervention in rural Liberia: A mixed-methods feasibility study
Source: PLoS One. 2024 Dec 13;19(12):e0311486. doi: 10.1371/journal.pone.0311486 (PMC11642910; doi:10.1371/journal.pone.0311486)
Supplement: S1 Method — (DOCX) [file pone.0311486.s001.docx]

**S1 Methods. Baseline qualitative interview guide for caregivers (Liberian English).**

**Thank you for participating in this interview. For this interview, we want would like to learn more about you and your child.**

[**1**] Tell me small thing about [child] and his/her behavior? What does he/she like to do?

**Now, let’s talk about [child’s] day.**

[**2**] Since this morning, what do you remember (child) doing and even yesterday morning up to bed time.

[**A**] Where did he/she go and with whom?

[**Probe**] All the people (anyone else?) at all the different locations listed. Prompt for time spent with elder siblings, father?

[**B**] What does [child] do in these different places and with these different people throughout the day? What kind of activities do they engage in in this location with this person? Anything else?

[**Probe**] All the activities with all the different people and locations listed in previous questions. Can give examples of activities such as mealtimes, bathing, talking, playing, reading, signing, going to the market or for a visit.

[**C**] Was this a regular thing?

[**3**] Of all the activities you just told me about which of these activities does [child] enjoy most? Tell me about why you think they enjoy this activity so much?

**Now, let’s talk small thing about some of the times you are with your child but you have to do small small works.**

[**4**] Tell me, what is like for you and your child? Are you able to talk with [child] when you are doing small small works?

**So we have talked about things your child does throughout the day and some of the things they do with you and their father. Now let’s talk about some of those hard times you can face (child).**

[**5**] If [child] is give you hard time, what you can do?

[**A**] For example, if your child cries and screams, what do you say or do?

[**B**] If the child purposely throws and breaks something, what do you do?

[**C**] Who in your family is mostly train the child?

[**D**] Compared to other children in the family or in other homes, is [child’s] behavior addressed differently? If yes, why?

[**Probe**] Prompt about gender if not mentioned.

[**6**] Tell us about your feeling in taking part in the workshop that teach you about to look after your children. Why do you have this feelings?

[**A**] If interested, what would be the best way to make it easier for you to attend the workshop?

[**Probe**] Reminders from community health assistant, someone to help attend to [child], distance from home, time of day.

[**B**] If you attended, what would you like to know about during these workshop? What types of activities, skills, or knowledge would you like to hear about?

[**7**] What else would you like to share about caring for your child that you expected to hear or think I should have asked you?

**Let’s talk about [child’s] food and eating.**

[**8**] Is the child eating sold food?

[**Probe**] Skip to sanitation and hygiene if the child is not eating solid food.

[**9**] What kind of food the child is eating?

[**10**] Why you decided to give this food to the child?

[**Probe**] Consistency, how food is cooked, what ingredients are used, child’s appetite, time to prepare, nutrients, taste, what family eats, cost, availability at home, availability in the market.

[**11**] Compared to your other children or the neighbor’s children, is your child given the same kind and amount of food? Why/why not?

[**Probe**] Differences because of child gender.

[**12**] What kinds of foods do you think your child needs to grow up healthy? Tell me why you think these foods are important?

**Now, let’s talk about some specific foods.**

[**13**] Do you mostly feed the child with chicken’s eggs?

[**A**] If not, why?

[**Probe**] Taboos, allergies, health concerns, expense, or child dislikes.

[**Probe**] If they say “expense/finance” –Would you give them to [child] if cost wasn’t an issue?

[**Probe**] If they say when older you can probe, “How much older?”

[**B**] If yes, why do you feed him/her egg? How do you typically prepare eggs for [child]?

[**14**] Do you mostly feed the child with fish?

[**A**] If not, why?

[**B**] If yes, why do you feed him/her fish? What types of fish (dried, fresh or frozen)? Is the fish mostly pounded or powdered? How do you prepare fish for [child] (for example, do you mix it in a dish or do you prepare it alone)?

[**15**] In your own thinking, how the food you give to your child helps them to grow health? What about to make brain fresh or think fast?

[**16**] What else do you think is important to know about feeding the child and the kind of food that I have not talked about but you still want to know.

[**17**] What can you tell me about handwashing in your house? When do you wash your hands?

[**Probe**] Other times.

[**18**] Tell me about how you wash your hands in your house?

[**Probe**] Soap or plain water.

[**19**] Why do you wash your hands at this time/these times?

[**NOTE**] Only ask this once.

**Now, let’s talk about some sicknesses.**

[**20**] Has the [child] ever had running stomach- happening more 3 times per day. If yes, when was the most recent time and what did you do?

[**21**] Has [child] ever had malaria? If yes, what did you do to take care of [child]? Why?

[**22**] Does [child] sleep under mosquito nets in your home? Why?

[**A**] If no, if somebody gave you one would you use it? If no, why not?

**Now let’s talk about your experiences with coronavirus.**

[**23**] What are doing for you to not to get corona virus?

[**Probe**] Face masks, social distancing, hand sanitizers, washing hands with soap and water, vaccination.

[**24**] What are you doing for your child to not get corona virus?

[**Probe**] Face masks, social distancing, hand sanitizers, washing hands with soap and water, vaccination.

[**25**] In your thinking, how is your child’s health?

[**A**] How often your child get sick? And how serious it can be?

[**B**] How this sickness is affect them to grow?

[**C**] How this sickness affect their brain to develop fresh or think fast?

[**26**] In your thinking, how does the food your child eats affect this sickness?

[**27**] Is there anything else that you would like to say about preventing or treating your child from sicknesses?

[**28**] Has Corona Virus changed the way you take care of your children? And How?

[**29**] What are some of hard time you have faced in taking care of your child during the corona?

[**30**] Which kind of feeling during the pandemic?

[**31**] How do you think your feeling changed when the corona virus started up to now?

[**32**] What kind help would you have liked from services (health, social etc.) during the period of the corona virus?

[**33**] Did you get the helps you wanted during the corona virus (for example, health, food, income etc.)?

[**34**] How easy or hard has it been to get the services for you and your child during the corona virus?

[**Probe**] Clinics, social services, financial services.

[**35**] Can you explain your experiences?

*Note. Interview guide is written in Liberian English.*
