# Supplementary material for: Integrated child nutrition, parenting, and health intervention in rural Liberia: A mixed-methods feasibility study
Source: PLoS One. 2024 Dec 13;19(12):e0311486. doi: 10.1371/journal.pone.0311486 (PMC11642910; doi:10.1371/journal.pone.0311486)
Supplement: S2 Method — (DOCX) [file pone.0311486.s002.docx]

**S2 Methods. Endline qualitative interview guide for caregivers (Liberian English).**

**Thank you for participating in this interview. For this interview, we want would like to learn more about you about your experience with the program. You were able to take part in the workshops with community health assistants to help your child grow healthy and help your child’s brain think fast.**

**You were able to take part in the workshops with community health workers to help your child grow healthy and make child’s brain fresh.**

[**1**] How did these workshops help you?

[**2**] What things did you like from the workshops? Why?

[**Probe**] Duration, frequency, location, delivery, content.

[**3**] What things did you not like from the workshops? Why?

[**Probe**] Duration, frequency, location, delivery, content.

[**4**] How do you think the workshops can improve?

[**Probe**] Participant’s responses from number 3.

[**5**] Did you have hard time taking part in the workshops?

[**A**] If so, why did you have hard time?

[**6**] Can you remember the messages from the workshop?

[**A**] If yes, explain what you remember.

[**7**] Now, let’s talk about the five messages. (*show caregiver list of 5 messages*)

[**A**] Were any of these messages important to you? If yes, why? If no, why not?

[**B**] Did you practice any of the messages outside of the workshops? If yes, how?

[**C**] Did this help you or your child?

[**D**] Which messages were easy for you to practice?

[**E**] Which messages were hard for you to practice?

[**8**] After the workshops, how is the way you play and talk with [child] different from before the workshops to now?

[**Probe**] Playthings, talking with child, reading materials.

[**9**] How do you think that the things you learned in these meetings can improve [child’s] brain, talk, or activity of [child]?

[**A**] Since the start of the workshops, have you seen a difference in [child’s] development compared to other children who are the same age?

[**10**] Do you think fathers should take part in the workshops with you or take part in different workshops for fathers only?

[**A**] Why/why not?

[**11**] Did you share what you learned from the workshops with [child’s] father, or members of your household, or other people in your community?

[**A**] If answer no: can you tell me why you didn’t share what you learned from the meetings with your family or neighbors?

[**B**] If answer yes: did they engage in new activities or behaviors with their children? If yes, how? If no, why not?

[**12**] Do you think it is important for the Community Health Workers to always come to your home to review the material you learned in the workshops?

[**A**] If yes, why?

[**B**] If no, why not?

**Now let’s talk about the eggs and fish you were given as part of the program?**

[**13**] Did you feed the child with eggs and fish?

[**A**] If yes, why? Did you feed both?

[**B**] If not, why?

[**14**] For every week, was the amount of eggs and fish enough to feed [child]?

[**Probe**] Feed only eggs or only fish, or both.

[**15**] Was the type of egg and fish okay for you and for [child]?

[**Probe**] Feed only eggs or only fish, or both.

[**16**] We gave you eggs and fish to feed the child and we gave you eggs and fish for the other people in house. Were the eggs and fish that we gave you for the child shared with other household members? If so, why?

[**Probe**] Feed only eggs or only fish, or both.

[**17**] Is there anything else that you would like to say that I have not talk about?

*Note. Interview guide is written in Liberian English.*
