# Supplementary material for: Integrated child nutrition, parenting, and health intervention in rural Liberia: A mixed-methods feasibility study
Source: PLoS One. 2024 Dec 13;19(12):e0311486. doi: 10.1371/journal.pone.0311486 (PMC11642910; doi:10.1371/journal.pone.0311486)
Supplement: S3 Method — (DOCX) [file pone.0311486.s003.docx]

# S3 Methods. Endline qualitative interview guide for Community Health Workers and general Community Health Volunteers (Liberian English).

**Thank you for participating in this interview. The purpose of this interview is to get information from you about your experience with the program.**

[**1**] Let’s begin by telling me what you do in your work?

[**2**] In your opinion, what are common challenges that caregivers in your community have about child’s growth?

[**A**] What are common challenges about their child’s development (for example, learning to talk, their brain growth, getting them ready for school)?

[**3**] In your opinion, which of these are more important issues that need to be addressed with caregivers (for example, time to play with child, income for nutritious food)?

[**4**] Over the past month, you lead a program aimed to help children’s development. What did you think of this parenting program?

**Before you started this study, you were trained on different parts of the program.**

[**5**] What do you think about the quality of training you received on the parenting program?

[**A**] What training activities did you find most useful and why?

[**B**] What training activities did you not find useful and why?

[**C**] What would you have liked to learn more about in these training sessions?

[**6**] Do you think you could have benefited from additional training?

[**A**] If yes, what additional training?

[**7**] Did you find the job aides and materials useful?

[**A**] What job aides and materials did you like and why?

[**B**] What job aides and materials did you not like and why?

[**8**] What is your opinion about the knowledge and skills shared with caregivers through the parenting program?

[**9**] In your opinion what did the caregivers think of the parenting program?

[**A**] What about other family members and other community members?

[**10**] Did you feel the knowledge and skills promoted through the parenting program were important to the caregivers in your community?

[**A**] If yes, what knowledge and skills were important and why?

[**B**] What knowledge and skills were promoted that were not important to the caregivers in your community?

[**C**] If no, why not?

[**11**] Were there any skills or activities which you think the caregivers used more? Or less?

[**A**] Why?

[**12**] Do you think that the knowledge and skills discussed were acceptable to the community?

[**13**] Do you think you could have benefitted from additional supervision or support?

[**A**] If yes, what additional support?

[**14**] Did this parenting program improve your knowledge and skills that you have learned previously?

[A] If yes, what aspects?

[**Probe**] Listening, communication skills, leading group discussion.

[**15**] Did you apply the knowledge and skills you learned from the parenting program in your own household?

[**A**] If yes, which aspects?

[**B**] If no, why not?

[**16**] Is this parenting program in line with the work that you usually do in your community as a community health assistant?

[**17**] Do you have any requests on how to improve this program?

[**A**] If yes, what?

## [Probe] Content, participation.

[**18**] Do you think fathers would attend these types of parenting sessions alone or with the female caregivers?

[**A**] Why/why not?

[**19**] In your opinion were the eggs and dried fish okay to children?

[**A**] If yes, why?

[**B**] If no, why not?

[**Probe**] If only eggs, only fish, or both.

[**20**] In your opinion was the amount of food and the weekly delivery of food enough?

[**A**] If no, why not?

[**21**] Do you have any requests on how the delivery of food could be improved?

[**A**] If yes, what are they?

[**22**] Did your time spent on the parenting program and delivering eggs and fish to households take away from your time to work as a community health assistant?

[**23**] Would you like to share anything else about the program?

*Note. Interview guide is written in Liberian English.*
