# Supplementary material for: Integrated child nutrition, parenting, and health intervention in rural Liberia: A mixed-methods feasibility study
Source: PLoS One. 2024 Dec 13;19(12):e0311486. doi: 10.1371/journal.pone.0311486 (PMC11642910; doi:10.1371/journal.pone.0311486)
Supplement: S1 Table — (DOCX) [file pone.0311486.s004.docx]

| **S1 Table. Responsive parenting sessions.** | |
| --- | --- |
| **Session Part 1: What kind of caregiver do you want to be?** | |
| Activities | Topics covered |
| 1. Caregiver hopes for child’s future | - Caregivers hopes for their children’s future (i.e., the type of person caregivers hope their child becomes) - Parenting practices that support children’s healthy growth and development |
| 1a. Challenges caregivers face | - Barriers or problems encountered when attempting to be ideal parent |
| 2. Feeding the brain with food and stimulation | - Five main parenting messages to support healthy growth and development: (1) love and respect, (2) two-way talk, (3) play, (4) hygiene and sanitation, (5) nutrition - Food diversity - Nutritious foods - Introducing foods (i.e., timing, quantity) - Healthy feeding practices |
| 3. Communication activity | - Two-way talk with child - Responsive interactions |
| 4. Final messages and encourage practice at home | - Five main parenting messages to support healthy growth and development: (1) love and respect, (2) two-way talk, (3) play, (4) hygiene and sanitation, (5) nutrition - Nutritious foods and healthy feeding practices - Practice five parenting messages - Practice two-way talk with child - Sharing information about parenting messages and nutrition with other household members |
| **Session Part 2: Provide a variety of stimulating objects for child’s play and talking with your child** | |
| Activities | Topics covered |
| 1. Practice play | - Play with child using two-way talk - Play with child using new objects - Child directed play - Importance of encouraging child directed play in relation to healthy cognitive development. |
| 2. Common problems to solve and discuss on play | - Importance of providing children with objects for play in relation to healthy cognitive development. - Objects to use as playthings - Hygiene and sanitation during play - Identifying time every day when child can play with some supervision |
| 3. Practice two-way talk with child about a picture | - New ways to talk with child - Two-way talk with images - Why talking is necessary - Responsive interaction |
| 4. Problem solving about talking with children | - Importance of providing child attention - Barriers to implementing two-way talk and play - Importance of adults engaging in two-way talk - Language development milestones |
| 5. Final messages and encourage practice at home | - Five main parenting messages to support healthy growth and development: (1) love and respect, (2) two-way talk, (3) play, (4) hygiene and sanitation, (5) nutrition - Practical objects to use for play - Practice play and two-way talk with child - Identify time every day when child can play with some supervision |
