# Supplementary material for: Integrated child nutrition, parenting, and health intervention in rural Liberia: A mixed-methods feasibility study
Source: PLoS One. 2024 Dec 13;19(12):e0311486. doi: 10.1371/journal.pone.0311486 (PMC11642910; doi:10.1371/journal.pone.0311486)
Supplement: S2 Table — (DOCX) [file pone.0311486.s005.docx]

| **S2 Table. Template for Intervention Description and Replication (TIDieR) checklist.** | |
| --- | --- |
| **Item name** | **Item Description** |
| 1. BRIEF NAME | **Combined child nutrition, parenting, and health intervention in rural Liberia: a feasibility study** |
| 1. WHY | Combined improvements in nutrition and responsive stimulation will benefit early childhood development.  **Nutrition supplementation:** improves child development through direct effects the brain and indirect effects through growth, reduced frequency and severity of illness, self-directed interaction with others, exploration of the environment.   - Animal-source foods: sources of nutrients essential for brain development; there is potential for scaling up. - Eggs: contains zinc, folate, riboflavin, choline, vitamins B6 and B12, protein, and docosahexaenoic acid. - Fish: contain protein, essential fatty acids, B vitamins, calcium, iron, and zinc, with minimal levels of contaminants (e.g., heavy metals) and microbial loads.   **Responsive stimulation (via responsive parenting sessions):** provides cognitive, language, and emotional support for age-appropriate development.   - Early learning activities*:* enhance caregivers’ access, knowledge, attitudes, and skills around supporting early learning. - Responsive parenting activities: encourage and support sensitivity and responsiveness. |
| 1. WHAT:   MATERIALS | **Nutrition supplementation** ***Training:*** CHWs and gCHVs received a manual describing the cooking demonstration for suggested preparation of the provided food. The manual also described the process and quantity of food to be delivered to participating caregivers. CHWs and gCHVs were trained over a one-week period by the study principal investigator, a graduate student, and study team members from Plan International Liberia. The training involved didactic sessions, practicing, and mock sessions.  ***Delivery:*** CHW distributed 14 eggs and 20 pieces of dried Bonny fish pieces to each participating caregiver, weekly.  **Responsive parenting sessions** ***Training:*** Community Health Workers (CHWs) and general Community Health Volunteer (gCHVs) facilitating the parenting sessions received a booklet of the responsive parenting manual. The manual detailed guidelines, processes to follow, important precautions, materials, and suggested scripts and dialogue for each session. CHWs and gCHVs were trained over a one-week period by the study principal investigator, a graduate student, and study team members from Plan International Liberia. The training involved didactic sessions, practicing, and mock sessions.  ***Delivery:*** In each community, facilitators (CHWs) received the following:   - Five-message poster and food groups poster to hang in a central location during each session. - Five-message handouts and food groups handouts to give to each caregiver. - Play bag with local readily-available objects specified in the manual to use as playthings for demonstration. - Two-way talk pictorials (for caregivers to use for activities and for the facilitator to use for demonstration). |
| 1. WHAT: PROCEDURES | **Nutrition supplementation**: Provision of eggs and dry Bonny fish. ***Demonstration:*** CHWs held a cooking demonstration to give suggestions to enrolled caregivers on how to prepare and feed the food to the child. The meal consisted of one egg, three pieces of dried Bonny fish pounded into dust, and fufu. ***Delivery:*** Weekly, at the central location where parenting sessions were held, CHW distributed seven eggs and ten pieces of dried Bonny fish to each participating caregiver for the participating child. The participating caregiver received an additional seven eggs and ten pieces of dried Bonny fish to provide to other household members and minimize sharing of the participating child’s food.  **Responsive parenting sessions**: Female caregivers and their children 6-36 months of age. ***Session topics:***  A: What kind of parent do you want to be? B: Stimulating objects for child's play and talking with children.  ***Procedure for each session:***  1. Welcome (CHW, gCHV, and caregivers introduce themselves); 2. gCHV takes attendance using the attendance ledger; 3. CHW states the purpose of the session; 4. Activities and discussion on the main message of the session; 5. Break out session for caregivers and children to participate in play and talk activities; 6. Q&A, open discussion of common problems and solutions for those problems; 7. Review main messages of the session, encourage at-home practice of activities; 8. Close session, inform of upcoming sessions; 9. [for CHWs only] Complete post-session review and submit to attending supervisor. |
| 1. WHO PROVIDED | **Nutrition supplementation and responsive parenting sessions**: Facilitators (n=2 in each community).  ***Background and expertise:*** Well-respected community members from each community, literate, and able to facilitate group sessions. CHWs are employed as part of the Liberia National Community Health Services Policy. They deliver a package of health activities to their communities, including referral and treatment of malaria, and health promotion messages around insecticide-treated bed net utilization, destruction of mosquito breeding sites, hygiene and cleaning of the home environment, and recognition of warning signs of malaria and other diseases. |
| 1. HOW | **Nutrition supplementation**: Weekly provision of food to caregivers at a central location in the community.  **Responsive parenting sessions**: Staggered group sessions with 7-8 caregiver-child dyads in each group. Total of 15 caregiver-child dyads per session. |
| 1. WHERE | **Nutrition supplementation**: Central location in each community.  **Responsive parenting sessions**: Central location in each community. |
| 1. WHEN and   HOW MUCH | **Intervention duration**: Four weeks. **Nutrition supplementation**: Provision of 14 eggs and 20 pieces of dry Bonny fish per week (seven eggs and ten pieces of fish for the participating child, seven eggs and ten pieces of fish provided for other household members to reduce sharing of food designated for the child).  **Responsive parenting session group size**: Fortnightly parenting sessions with female and male caregivers of children 6-36 months of age. Sessions were held separately in each community and staggered to accommodate a smaller group size (7-8 caregiver-child dyads). **Nutrition supplementation and responsive parenting sessions schedule**:   \| **Week 1** \| ***Day 1***: (groups I & II) cooking demonstration \| \| --- \| --- \| \|  \| ***Day 2***: (group I) seven caregiver-child dyads from each community attend parenting session A; provision of eggs and fish (post-session) \| \|  \| ***Day 3***: (group II) eight caregiver-child dyads from each community attend parenting session A; provision of eggs and fish (post-session) \| \| **Week 2** \| ***Day 1***: (groups I & II) provision of eggs and fish \| \| **Week 3** \| ***Day 1***: (group I) seven caregiver-child dyads from each community attend parenting session B; provision of eggs and fish (post-session) \| \|  \| ***Day 2***: (group II) eight caregiver-child dyads from each community attend parenting session B; provision of eggs and fish (post-session) \| \| **Week 4** \| ***Day 1***: (groups I & II) provision of eggs and fish \| |
| 1. TAILORING | **Adaptations made to intervention to fit local context**:   - Images in the five-message poster reflect the culture. - Images in the food groups poster reflect the locally available foods. - Playthings used in demonstrations are typical of objects found in the local community (not store-bought). - Typical language and speech patterns in the responsive parenting manual fit local dialects. - Responsive parenting sessions are scheduled to accommodate caregivers' schedules. - Central location for intervention selected to avoid travel-related issues and accommodate caregivers' circumstances. |
| 1. MODIFICATIONS | **Training facilitators**:   - Find facilitators in the community who have the necessary skills to facilitate sessions, for instance, public speaking, confidence, material comprehension, patience, motivation and interest in intervention, trustworthiness. - Train two facilitators in each community and have backup facilitators to watch children during discussion portions of sessions. - Develop a detailed schedule of responsibilities for each role.   **Delivery**:   - Inform community leader of each session, request sessions are private, with minimal distractions from community members. - Minimize distraction from children when the caregivers need to pay attention to the session; find appropriate location for children to play with and be cared for by the gCHV to minimize distraction. |
| 1. HOW WELL: PLANNED | - Rigorous training of facilitators consisting of reviewing material (i.e., discussion and activities in each session followed by mock sessions led by the CHW). - Rigorous assessment of competencies to deliver the program. |
| 1. HOW WELL:   ACTUAL | - Participation logs to track caregiver attendance. - Post-session report completed by facilitators after each session. - Supervision and monitoring of intervention by elected staff members from the study team organization (Plan International Liberia). - Regardless of experience or knowledge of early child development, staff members attend all training meetings on intervention and parenting sessions. |
| CHW: Community Health Worker; gCHV: general Community Health Volunteer | |
