# Supplementary material for: Integrated child nutrition, parenting, and health intervention in rural Liberia: A mixed-methods feasibility study
Source: PLoS One. 2024 Dec 13;19(12):e0311486. doi: 10.1371/journal.pone.0311486 (PMC11642910; doi:10.1371/journal.pone.0311486)
Supplement: S3 Table — (DOCX) [file pone.0311486.s006.docx]

| **S3 Table. Demographic characteristics of study participants at baseline.** | | | | | |
| --- | --- | --- | --- | --- | --- |
| **Characteristics** | | | | **Number/Number of Responses (%) or Mean +/- SD** | |
| **Sociodemographic** | | | | |  |
|  | Caregiver age (years) | | | |  |
|  |  | 18-24 | | | 12/30 (40) |
|  |  | 25-34 | | | 8/30 (26.7) |
|  |  | 35-44 | | | 7/30 (23.3) |
|  |  | 45-50 | | | 3/30 (10) |
|  |  | Mean caregiver age; min = 18, max = 50 | | | 29.8 +/- 9.9 |
|  | Child age (months) | | | |  |
|  |  | 6-11 | | | 9/30 (30) |
|  |  | 12-23 | | | 11/30 (36.7) |
|  |  | 24-36 | | | 10/30 (33.3) |
|  |  | Mean child age; min = 6, max = 35 | | | 18.1 +/- 9.1 |
|  | Female child | | | | 14/30 (46.7) |
|  | Religion | | | |  |
|  |  | Christian | | | 22/30 (73.3) |
|  |  | Islam | | | 8/30 (26.7) |
|  | Female caregiver: completed grade | | | |  |
|  |  | Never attended | | | 17/30 (56.7) |
|  |  | Sixth grade and under | | | 6/30 (20) |
|  |  | Twelfth grade and under | | | 7/30 (23.3) |
|  | Female caregiver: occupation | | | |  |
|  |  | Unemployed | | | 2/30 (6.7) |
|  |  | Irregular job (day labor, unskilled worker, labor who manage household affairs, rickshaw puller/ van puller, fisherman, farmer who cultivate other’s land, hawker, etc.) | | | 1/30 (3.3) |
|  |  | Regular job (farmer who cultivate own land, private business, shopkeeper, private job, skilled worker, skilled garment worker, office staff, salesman, government official) | | | 27/30 (90) |
|  | Father: completed grade | | | |  |
|  |  | Never attended | | | 16/30 (53.3) |
|  |  | Sixth grade and under | | | 1/30 (3.3) |
|  |  | Twelfth grade and under | | | 13/30 (43.3) |
|  | Father: occupation | | | |  |
|  |  | Unemployed | | | 0/30 (0) |
|  |  | Irregular job (day labor, unskilled worker, labor who manage household affairs, rickshaw puller/ van puller, fisherman, farmer who cultivate other’s land, hawker, etc.) | | | 7/30 (23.3) |
|  |  | Regular job (farmer who cultivate own land, private business, shopkeeper, private job, skilled worker, skilled garment worker, office staff, salesman, government official) | | | 23/30 (76.7) |
|  | Total members of household; min = 3, max = 16 | | | | **7.3 +/- 3.0** |
|  | Number children under 5 years of age in household; min = 1, max = 4 | | | | 1.9 +/- 1.1 |
|  | Total of your children; min = 1, max = 7 | | | | 3.8 +/- 2.2 |
|  | Birth order of child; min = 1, max = 7 | | | | 3.2 +/- 2.1 |
|  | Monthly family income (Liberian dollars) | | | |  |
|  |  | <10,000 | | | 17/30 (56.7) |
|  |  | 10,000-<50,000 | | | 13/30 (43.3) |
|  | Sufficient money | | | |  |
|  |  | Never | | | 3/30 (10) |
|  |  | Occasionally | | | 27/30 (90) |
|  |  | Always | | | 0/30 (0) |
|  | Housing (rent or own) | | | |  |
|  |  | Rent a house | | | 6/30 (20) |
|  |  | Own a house | | | 20/30 (66.7) |
|  |  | Family house | | | 4/30 (13.3) |
|  | Number of rooms in house; min = 2, max = 7 | | | | 3.8 +/- 1.2 |
|  | Electricity | | | | 0/30 (0) |
|  | Possess land | | | | 26/30 (86.7) |
|  | Roof materials | | | |  |
|  |  | Rudimentary roof (palm/bamboo, wood planks, plastic) | | | 1/30 (3.3) |
|  |  | Finished roof (metal, wood, cement, etc.) | | | 29/30 (96.7) |
|  | Floor materials | | | |  |
|  |  | Natural floor (mud, bamboo) | | | 18/30 (60) |
|  |  | Finished floor (metal, wood, cement, etc.) | | | 12/30 (40) |
|  | Wall materials | | | |  |
|  |  | Natural wall (mud, thatch/palm leaf/sod) | | | 26/30 (86.7) |
|  |  | Finished (metal, wood, cement, etc.) | | | 4/30 (13.3) |
|  | Cooking fuel materials | | | |  |
|  |  | Charcoal/coal-like matter/wooden coal | | | 5/30 (16.7) |
|  |  | Wood | | | 25/30 (56.7) |
|  | Livestock: duck or hen; min = 0, max = 15 | | | | 3.3 +/- 3.8 |
|  | Total livestock; min = 0, max = 18 | | | | 3.4 +/- 4.2 |
|  | Breastfeeding currently among children <24 months | | | | 12/20 (60) |
| **Insecticide-treated nets (ITN)** | | | | |  |
|  | Household has ITN | | | | 23/30 (76.7) |
|  | *The following questions were answered by those who have ITN (n=23)* | | | |  |
|  | Number of ITNs; min = 1, max = 9 | | | | 2.3 +/- 1.9 |
|  | ITN age | | | |  |
|  |  | Don't know/not sure | | | 20/23 (87) |
|  | Net obtained from | | | |  |
|  |  | Non-governmental organization | | | 4/23 (17.4) |
|  |  | Mass distribution campaign | | | 1/23 (4.4) |
|  |  | Government health facility | | | 16/23 (69.6) |
|  |  | Shop / market / street | | | 1/23 (4.4) |
|  |  | Community health worker | | | 1/23 (4.4) |
|  | Net used last night (yes) | | | | 5/23 (21.7) |
|  |  | Among those who used the nets last night, how many people slept under the ITN used last night? min = 3, max = 10 | | | 5 +/- 2.9 |
| **Water, Sanitation, and Hygiene** | | | | |  |
|  | Main source of drinking water for members of household | | | |  |
|  |  | Tubewell/borehole (improved drinking source) | | | 24/30 (80) |
|  |  | Surface water (river, dam, lake, pond, stream, canal, irrigation channels)  (no drinking water facilities) | | | 6/30 (20) |
|  | Treat water in any way to make it safe to drink | | | | 21/30 (70) |
|  | Among those who treat their water (n=21), what is done to make it safe? | | | |  |
|  |  | Add bleach/chlorine | | | 6/21 (28.6) |
|  |  | Let it stand and settle | | | 15/21 (71.4) |
|  | Main source of water for other purposes (cooking and hand washing) | | | |  |
|  |  | Tubewell/borehole | | | 5/30 (16.7) |
|  |  | Surface water (river, dam, lake, pond, stream, canal, irrigation channels) | | | 25/30 (83.3) |
|  | Type of toilet facility | | | |  |
|  |  | Piped sewer system (improved sanitation facility) | | | 1/30 (3.3) |
|  |  | Pit latrine (unimproved sanitation facility) | | | 3/30 (10) |
|  |  | No facilities or bush or field (no sanitation facility) | | | 26/30 (86.7) |
|  | Share toilet with other households | | | | 13/30 (43.3) |
|  | Last time child passed stools--how was this disposed of? | | | |  |
|  |  | Child used toilet/latrine | | | 0/30 (0) |
|  |  | Put/rinsed into toilet or latrine | | | 2/30 (6.7) |
|  |  | Put/rinsed into drain or ditch | | | 14/30 (46.7) |
|  |  | Thrown into garbage | | | 7/30 (23.3) |
|  |  | Buried | | | 7/30 (23.3) |
|  | Important to wash hands: before eating (yes) | | | | 21/30 (70) |
|  | Important to wash hands: before feeding child (yes) | | | | 24/30 (80) |
|  | Important to wash hands: before cooking/preparing food (yes) | | | | 27/30 (90) |
|  | Important to wash hands: after urination/defecation (yes) | | | | 29/30 (96.7) |
|  | Important to wash hands: cleaning child who has urinated/defecated (yes) | | | | 24/30 (80) |
|  |  | Wash hands with: soap and water | | | 30/30 (100) |
|  | Human feces around home or in compound (yes) | | | | 2/30 (6.7) |
|  | Animal feces around home or in compound (yes) | | | | 7/30 (23.3) |
|  | Garbage around home or in compound (yes) | | | | 2/30 (6.7) |
| **Food insecurity** | | | | |  |
|  | Worry household doesn't have enough food | | | | 29/30 (96.7) |
|  | You/household members not able to eat kinds of food preferred due to lack of resources | | | | 30/30 (100) |
|  | You/household members limited variety of foods due to lack of resources | | | | 28/30 (93.3) |
|  | You/household members eat foods did not want to eat due to lack of resources | | | | 29/30 (96.7) |
|  | You/household members eat smaller meals not enough food | | | | 28/30 (93.3) |
|  | You/household members eat fewer meals not enough food | | | | 28/30 (93.3) |
|  | No food to eat due to lack of resources | | | | 12/30 (40) |
|  | You/household members sleep hungry due to not enough food | | | | 2/30 (6.7) |
|  | You/household members go a whole day without eating due to not enough food | | | | 2/30 (6.7) |
|  | Food insecurity score (range: 0-27); min = 0, max = 18  (higher score indicates higher food insecurity (access)) | | | | 11.2 +/- 3.8 |
| **Maternal psychological distress** | | | | |  |
|  | Psychological distress score (range 0-20); min = 0, max = 8  (higher score indicates worse psychological distress) | | | | 2.9 +/- 2.3 |
| **Child discipline** | | | | |  |
|  | You or another adult in the household disciplines child with taking away privileges, forbidding something the child liked, or did not allow him/her to leave the house | | | | 1/30 (3.3) |
|  | You or another adult in the household disciplines child with explaining why the behavior was wrong | | | | 29/30 (96.7) |
|  | You or another adult in the household disciplines child with shaking him/her | | | | 1/30 (3.3) |
|  | You or another adult in the household disciplines child with shouting, yelling, or screaming at him/her | | | | 13/30 (43.3) |
|  | You or another adult in the household disciplines child with giving him/her something else to do | | | | 30/30 (100) |
|  | You or another adult in the household disciplines child with spanking, hitting, or slapping him/her on the bottom with bare hand | | | | 9/30 (30) |
|  | You or another adult in the household disciplines child with hitting him/her on the bottom or elsewhere on the body with something like a belt, hairbrush, stick, or other hard object | | | | 5/30 (16.7) |
|  | You or another adult in the household disciplines child with calling him/her dumb, lazy, or another name like that | | | | 6/30 (20) |
|  | You or another adult in the household disciplines child with hitting or slapping him/her on the face, head, or ears | | | | 0/30 (0) |
|  | You or another adult in the household disciplines child with hitting or slapping him/her on the hand, arm, or leg | | | | 10/30 (33.3) |
|  | You or another adult in the household disciplines child with beating him/her up, that is hit him/her over and over as hard as one could | | | | 0/30 (0) |
|  | You believe that in order to bring up, raise, or educate a child properly, the child needs to be physically punished | | | | 4/30 (13.3) |
|  | Discipline score (range: 0-12); min = 2, max = 8  (a higher score indicates worse discipline actions by caregiver) | | | | 3.6 +/- 1.3 |
| **Child dietary diversity** | | | | |  |
|  | Ever breastfed | | | | 29/30 (96.7) |
|  | Breastfed yesterday (children <24 months) | | | | 13/20 (65) |
|  | Meets minimum meal frequency (among children <24 months) | | | | 12/20 (60) |
|  | Meets minimum dietary diversity (≥5 of 8 food groups) | | | | 24/30 (80) |
|  | Child dietary diversity score (range 0-8); min = 1, max = 8 | | | | 5.5 +/- 2.0 |
| **Child health** | | | | |  |
|  | Diarrhea in the past 2 weeks | | | | 9/30 (30) |
|  | Fever in the past 2 weeks | | | | 17/30 (56.7) |
|  | Cough in the past 2 weeks | | | | 16/30 (53.3) |
|  | Difficulty breathing in the past 2 weeks | | | | 0/30 (0) |
| **Responsive feeding (in the past 2 weeks)** | | | | |  |
|  | Child takes bite | | | |  |
|  |  | Almost never (0-1 days/week) | | | 8/30 (26.7) |
|  |  | Occasionally (2-3 days/week) | | | 8/30 (26.7) |
|  |  | Most days (4-5 days/week) | | | 0/30 (0) |
|  |  | Almost every day | | | 14/30 (46.7) |
|  | Child refuses to eat | | | |  |
|  |  | Almost never (0-1 days/week) | | | 15/30 (50) |
|  |  | Occasionally (2-3 days/week) | | | 15/30 (50) |
|  |  | Most days (4-5 days/week) | | | 0/30 (0) |
|  |  | Almost every day | | | 0/30 (0) |
|  | Pressure child to eat | | | |  |
|  |  | Almost never (0-1 days/week) | | | 21/30 (70) |
|  |  | Occasionally (2-3 days/week) | | | 9/30 (30) |
|  |  | Most days (4-5 days/week) | | | 0/30 (0) |
|  |  | Almost every day | | | 0/30 (0) |
|  | Trick child to eat | | | |  |
|  |  | Almost never (0-1 days/week) | | | 20/30 (66.7) |
|  |  | Occasionally (2-3 days/week) | | | 9/30 (30) |
|  |  | Most days (4-5 days/week) | | | 0/30 (0) |
|  |  | Almost every day | | | 1/30 (3.3) |
|  | Praise child when eating | | | |  |
|  |  | Almost never (0-1 days/week) | | | 17/30 (56.7) |
|  |  | Occasionally (2-3 days/week) | | | 11/30 (36.7) |
|  |  | Most days (4-5 days/week) | | | 0/30 (0) |
|  |  | Almost every day | | | 2/30 (6.7) |
|  | When eating, child is positioned to see their face | | | |  |
|  |  | Almost never (0-1 days/week) | | | 10/30 (33.3) |
|  |  | Occasionally (2-3 days/week) | | | 7/30 (23.3) |
|  |  | Most days (4-5 days/week) | | | 1/30 (3.3) |
|  |  | Almost every day | | | 12/30 (40) |
|  | Child stays seated while eating | | | |  |
|  |  | Almost never (0-1 days/week) | | | 9/30 (30) |
|  |  | Occasionally (2-3 days/week) | | | 8/30 (26.7) |
|  |  | Most days (4-5 days/week) | | | 0/30 (0) |
|  |  | Almost every day | | | 13/30 (43.3) |
|  | Let child decide what to eat | | | |  |
|  |  | Almost never (0-1 days/week) | | | 24/30 (80) |
|  |  | Occasionally (2-3 days/week) | | | 5/30 (16.7) |
|  |  | Most days (4-5 days/week) | | | 1/30 (3.3) |
|  |  | Almost every day | | | 0/30 (0) |
| **Early learning opportunities** | | | | |  |
|  | Child has things for moving around | | | | 20/30 (66.7) |
|  | Child has things for role playing | | | | 21/30 (70) |
|  | Child has things for manipulation | | | | 9/30 (30) |
|  | Child has things that make sound | | | | 26/30 (86.7) |
|  | Child has things like a picture book | | | | 2/30 (6.7) |
|  | Child has things that are colorful | | | | 1/30 (3.3) |
|  | Child has area for playthings | | | | 7/30 (23.3) |
|  | Read to child | | | | 1/30 (3.3) |
|  | Take child out | | | | 26/30 (86.7) |
|  | Tell child stories | | | | 1/30 (3.3) |
|  | Sing to child | | | | 23/30 (76.7) |
|  | Child has structured games | | | | 16/30 (53.3) |
|  | Talk to child | | | | 21/30 (70) |
|  | Give child new playthings | | | | 8/30 (26.7) |
|  | Early learning score (range 0-14); min = 1, max = 10  (higher indicates better early learning environment) | | | | 6.2 +/- 2.3 |
| **Child development** | | | | |  |
|  | Overall child development score (range: 0-108); min = 0, max = 55 | | | | 26.1 +/- 16.9 |
|  | Motor domain score (range: 0-40); min = 0, max = 30 | | | | 15.3 +/- 9.7 |
|  | Cognition domain score (range: 0-32); min = 0, max = 16 | | | | 5.9 +/- 4.9 |
|  | Language domain score (range: 0-39); min = 0, max = 11 | | | | 3.3 +/- 3.8 |
|  | Social emotional domain score (range: 0-23); min = 0, max = 11 | | | | 5.2 +/- 2.7 |
| **Child mental health** | | | | |  |
|  | Child mental health score (range 0-9); min = 1, max = 6  (higher score indicates worse mental health) | | | | 2.5 +/- 1.3 |
| **COVID-19** | | |  | |  |
| **Caregiver section** | | | | |  |
|  | Since start of pandemic, how has your life changed | | | |  |
|  |  | Improved | | | 21/29 (72.4) |
|  |  | Same | | | 8/29 (27.6) |
|  |  | Worsened | | | 0/29 (0) |
|  | A year from now, how do you expect your life will change | | | |  |
|  |  | Better | | | 29/29 (100) |
|  |  | Same | | | 0/29 (0) |
|  |  | Worsened | | | 0/29 (0) |
|  | How have each of the following changed since the start of the COVID-19 pandemic: | | | | |
|  | Physical health | | | |  |
|  |  | Improved | | | 24/29 (82.8) |
|  |  | Same | | | 5/29 (17.2) |
|  |  | Worsened | | | 0/29 (0) |
|  | Illness frequency | | | |  |
|  |  | Improved | | | 25/29 (86.2) |
|  |  | Same | | | 4/29 (13.8) |
|  |  | Worsened | | | 0/29 (0) |
|  | Weight | | | |  |
|  |  | Gained | | | 18/29 (62.1) |
|  |  | Same | | | 8/29 (27.6) |
|  |  | Lost | | | 3/29 (10.3) |
|  | Physical activity | | | |  |
|  |  | More | | | 18/29 (62.1) |
|  |  | Same | | | 11/29 (37.9) |
|  |  | Less | | | 0/29 (0) |
|  | Sleep | | | |  |
|  |  | Improved | | | 17/29 (58.6) |
|  |  | Same | | | 12/29 (41.4) |
|  |  | Worsened | | | 0/29 (0) |
| **Household** | | |  | |  |
|  | How has each of the following changed since the start of the COVID-19 pandemic: | | | |  |
|  | Amount of childcare responsibilities | | | |  |
|  |  | More | | | 26/29 (89.7) |
|  |  | Same | | | 2/29 (6.9) |
|  |  | Less | | | 1/29 (3.5) |
|  | Responsiveness and attentiveness towards child | | | |  |
|  |  | Improved | | | 28/29 (96.6) |
|  |  | Same | | | 1/29 (3.5) |
|  |  | Worsened | | | 0/29 (0) |
|  | Amount of play activities with child | | | |  |
|  |  | More | | | 26/29 (89.7) |
|  |  | Same | | | 2/29 (6.7) |
|  |  | Less | | | 1/29 (3.5) |
|  | Temper with child | | | |  |
|  |  | Improved | | | 25/29 (86.2) |
|  |  | Same | | | 3/29 (10.3) |
|  |  | Worsened | | | 1/29 (3.5) |
|  | Harshness of discipline | | | |  |
|  |  | More | | | 1/29 (3.5) |
|  |  | Same | | | 0/29 (0) |
|  |  | Less | | | 28/29 (96.6) |
| **Economic circumstances** | | | | |  |
|  | Since the start of the COVID-19 pandemic, have you or anyone else in the household: | | | | |
|  | Lost income or salary | | | | 19/29 (65.5) |
|  | Seen income or salary decrease | | | | 18/29 (62.1) |
|  | Lost work or had to reduce hours | | | | 19/29 (65.5) |
|  | Left school and not able to return | | | | 16/29 (55.2) |
|  | Stayed sick for long / couldn't afford treatment | | | | 27/29 (93.1) |
|  | Amount of money the household borrows changed | | | |  |
|  |  | Increased | | | 1/29 (3.5) |
|  |  | Same | | | 3/29 (10.3) |
|  |  | Decreased | | | 25/29 (86.2) |
| **Food security** | | | | |  |
|  | How has each of the following changed since the start of the COVID-19 pandemic: | | | |  |
|  | Ability to find and pay for food | | | |  |
|  |  | Increased | | | 10/29 (34.5) |
|  |  | Same | | | 12/29 (41.4) |
|  |  | Decreased | | | 7/29 (24.1) |
|  | Availability of foods at home | | | |  |
|  |  | Increased | | | 7/29 (24.1) |
|  |  | Same | | | 13/29 (44.8) |
|  |  | Decreased | | | 9/29 (31) |
|  | Availability of foods at market | | | |  |
|  |  | Increased | | | 11/29 (37.9) |
|  |  | Same | | | 11/29 (37.9) |
|  |  | Decreased | | | 7/29 (24.1) |
|  | Price of foods | | | |  |
|  |  | Increased | | | 26/29 (89.7) |
|  |  | Same | | | 2/29 (6.9) |
|  |  | Decreased | | | 1/29 (3.5) |
|  | Quality of foods | | | |  |
|  |  | Increased | | | 9/29 (31) |
|  |  | Same | | | 12/29 (41.4) |
|  |  | Decreased | | | 8/29 (27.6) |
| **Child** | | |  | |  |
|  | Child experienced loss of caregiver or parent | | | | 1/29 (3.5) |
|  | Child experienced sickness of caregiver or parent | | | | 2/29 (6.9) |
|  | Behavior problems | | | |  |
|  |  | Increased | | | 16/29 (55.2) |
|  |  | Same | | | 7/29 (24.1) |
|  |  | Decreased | | | 6/29 (20.7) |
|  | Emotional difficulties | | | |  |
|  |  | Increased | | | 10/29 (34.5) |
|  |  | Same | | | 10/29 (34.5) |
|  |  | Decreased | | | 9/29 (31) |
|  | Physical health | | | |  |
|  |  | Improved | | | 27/29 (93.1) |
|  |  | Same | | | 2/29 (6.9) |
|  |  | Worsened | | | 0/29 (0) |
|  | Illness frequency | | | |  |
|  |  | Improved | | | 25/29 (86.2) |
|  |  | Same | | | 4/29 (13.8) |
|  |  | Worsened | | | 0/29 (0) |
|  | Weight | | | |  |
|  |  | Gained | | | 26/29 (89.7) |
|  |  | Same | | | 3/29 (10.3) |
|  |  | Lost | | | 0/29 (0) |
|  | Physical activity | | | |  |
|  |  | More | | | 27/29 (93.10) |
|  |  | Same | | | 2/29 (6.9) |
|  |  | Less | | | 0/29 (0) |
|  | Amount of time child plays outside | | | |  |
|  |  | More | | | 24/29 (82.8) |
|  |  | Same | | | 4/29 (13.8) |
|  |  | Less | | | 1/29 (3.5) |
|  | Amount of time child plays with others | | | |  |
|  |  | More | | | 24/29 (82.8) |
|  |  | Same | | | 3/29 (10.4) |
|  |  | Less | | | 2/29 (6.9) |
|  | Sleep | | | |  |
|  |  | Improved | | | 21/29 (72.4) |
|  |  | Same | | | 7/29 (24.1) |
|  |  | Worsened | | | 1/29 (3.5) |
|  | Amount/quantity of food | | | |  |
|  |  | More | | | 27/29 (93.1) |
|  |  | Same | | | 2/29 (6.9) |
|  |  | Less | | | 0/29 (0) |
|  | Variety/quality of food | | | |  |
|  |  | Improved | | | 24/29 (82.8) |
|  |  | Same | | | 5/29 (17.2) |
|  |  | Worsened | | | 0/29 (0) |
|  | Child born during pandemic | | | | 13/29 (44.8) |
|  | Think breastfeeding is safe in pandemic | | | | 20/29 (69) |
|  | Nurse at health facility provided information about breastfeeding during pandemic | | | | 23/29 (79.3) |
| **Risk mitigation** | | | | |  |
|  | Believe the COVID-19 pandemic is real (yes) | | | | 22/29 (75.9) |
|  | Since the beginning of the pandemic, what preparation/responses have you done for the emergency: | | | | |
|  | COVID response 0: do nothing | | | | 26/29 (89.7) |
|  | COVID response 1: wash hands regularly with soap and water | | | | 29/29 (100) |
|  | COVID response 2: disinfect surfaces and cleaning | | | | 16/29 (55.2) |
|  | COVID response 3: keeping distance from sick people | | | | 20/29 (69) |
|  | COVID response 4: keeping physical distance from everyone that is not a member of household | | | | 19/29 (65.5) |
|  | COVID response 5: stopped going to social gatherings and religious services | | | | 19/29 (65.5) |
|  | COVID response 6: wearing a face mask | | | | 29/29 (100) |
|  | COVID response 7: stocking up on food, supplies, medicine | | | | 0/29 (0) |
|  | COVID response 8: changing/canceling travel plans | | | | 0/29 (0) |
|  | If someone was sick in family, able to isolate at home | | | | 21/29 (72.4) |
|  | In public places, able to socially distance from other people | | | | 27/29 (93.1) |
|  | Able to find and obtain soap or hand sanitizer for yourself and family | | | | 29/29 (100) |
|  | Access to clean water for handwashing in compound | | | | 29/29 (100) |
|  | Think you could find and obtain and COVID test | | | | 24/29 (82.8) |
| **Moving forward** | | | | |  |
|  | When a COVID vaccine becomes available, you and your family will get vaccinated (yes) | | | | 24/29 (82.8) |
