# Supplementary material for: Integrated child nutrition, parenting, and health intervention in rural Liberia: A mixed-methods feasibility study
Source: PLoS One. 2024 Dec 13;19(12):e0311486. doi: 10.1371/journal.pone.0311486 (PMC11642910; doi:10.1371/journal.pone.0311486)
Supplement: S4 Table — (DOCX) [file pone.0311486.s007.docx]

| **S4 Table. Results from quantitative endline surveys.** | | | | |
| --- | --- | --- | --- | --- |
| **Variables** | | | **Number/Number of Responses (%) or Mean +/- SD** | |
| **Nutrition supplementation** | | | |  |
|  | Fed child at least one egg in the past week | | | 29/29 (100) |
|  | How many times in the past week fed child eggs provided | | |  |
|  |  | Less than 3 times/week | | 5/29 (17.4) |
|  |  | 3 times/week | | 17/29 (58.6) |
|  |  | 7 times/week | | 7/29 (24.2) |
|  | How much of the egg did the child eat each time | | |  |
|  |  | Only part of the egg | | 0/29 (0) |
|  |  | All of the egg | | 29/29 (100) |
|  | How did you prepare the egg for the child | | |  |
|  |  | Fried | | 9/29 (31) |
|  |  | Boiled | | 14/29 (48.3) |
|  |  | Mix with fufu | | 3/29 (10.3) |
|  |  | Mix with rice and Bonny and fry | | 1/29 (3.5) |
|  |  | Mix with fish and eddoes | | 1/29 (3.5) |
|  |  | Both fried and boiled (sometimes one or the other) | | 1/29 (3.5) |
|  | Egg never shared with others | | | 29/29 (100) |
|  | Fed child at least one fish in the past week | | | 29/29 (100) |
|  | How many times in the past week fed child fish provided | | |  |
|  |  | Less than 3 times/week | | 8/29 (27.6) |
|  |  | 3 times/week | | 14/29 (48.3) |
|  |  | 7 times/week | | 7/29 (24.1) |
|  | How much of the fish (in pieces) did child eat each time | | | 1.8 +/- 0.7 |
|  | How did you prepare the fish for the child | | |  |
|  |  | Mix with fufu | | 15/29 (51.7) |
|  |  | Mix with rice | | 9/29 (31) |
|  |  | Mix with any food | | 5/29 (17.2) |
|  | Type of fish was acceptable (yes) | | | 29/29 (100) |
|  | Fish never shared with others | | | 29/29 (100) |
| **Responsive parenting sessions** | | | |  |
|  | Number of sessions attended | | |  |
|  |  | One session | | 1/29 (3.5) |
|  |  | Two sessions (all) | | 28/29 (96.5) |
|  | Interested in attending more sessions if had the opportunity | | |  |
|  |  | Yes, very much | | 29/29 (100) |
|  |  | Somewhat | | 0/29 (0) |
|  |  | Not at all | | 0/29 (0) |
|  | Recalled any messages received over the past month (yes) | | | 28/29 (96.6) |
|  | Number of messages recalled | | |  |
|  |  | 0 messages | | 1/29 (3.5) |
|  |  | 4 messages | | 1/29 (3.5) |
|  |  | 5 messages (all) | | 27/29 (93.1) |
|  | Recalled message 1: Feed your child animal-source foods, 3 meals + 2 snacks, 4 to 8 handfuls of cooked food at each meal | | | 28/29 (96.6) |
|  | Recalled message 2: Parents wash their own and children’s hands with soap and water before touching food and after latrine use | | | 28/29 (96.6) |
|  | Recalled message 3: Provide your child with a variety of stimulating play objects | | | 28/29 (96.6) |
|  | Recalled message 4: Parents talk and sing with children, watch and listen to their child’s sounds | | | 28/29 (96.6) |
|  | Recalled message 5: Parents show love and respect for themselves, their children and their partner | | | 27/29 (93.1) |
|  | Able to do any of the following: feed child animal-source foods, 3 meals + 2 snacks, 4-8 handfuls of cooked food at each meal | | | 28/29 (96.6) |
|  |  | Able to do: (1) Feed child animal-source foods | | 24/29 (82.8) |
|  |  | Able to do: (2) Feed child 3 meals + 2 snacks every day | | 25/29 (86.2) |
|  |  | Able to do: (3) Feed child 4-8 handfuls of cooked food at each meal | | 23/29 (79.3) |
|  | Able to wash hands (and child's) with soap and water before touching food and after latrine use | | | 29/29 (10) |
|  | Able to provide child with stimulating play objects | | | 29/29 (100) |
|  | Able to sing and talk with children and watch and listen to child's sounds | | | 29/29 (100) |
|  | Able to show love and respect to your partner and children | | | 29/29 (100) |
